# Sugars, Salt, Saturated Fat and Fibre Purchased through Packaged Food and Soft Drinks in Europe 2015–2018: Are We Making Progress?

**DOI:** 10.3390/nu13072416

**Published:** 2021-07-14

**Authors:** Maria Alice Moz-Christofoletti, Jan Wollgast

**Affiliations:** Joint Research Center, European Commission, 21027 Ispra, Italy; maria.moz-christofoletti@ec.europa.eu

**Keywords:** nutrient content, food offer, food supply

## Abstract

The availability, purchase and consumption of foods high in fat, sugars and salt and low in fibre are linked to the high health and economic burden of noncommunicable diseases, including cancer, in Europe. Therefore, assessing the quality of the food offer is key as feedback to decision makers, as well as to identify good practices and areas of the food supply still requiring urgent action. We combined detailed market share and sales data with nutrition composition data to evaluate the nutritional quality of 14 packaged food and soft drinks categories sold across 22 European countries over the 2015–2018 period. Our analysis shows great variability of the nutritional composition within and among packaged food and soft drinks categories across European countries. Our estimates of the market-share weighted mean, a measure that integrates possible changes in nutrient content with the amount of a product sold to consumers, as well as daily per capita nutrient sale estimates, suggest a small but statistically significant progress in certain food categories only. Overall, the amounts of sugars, saturated fat, salt and fibre being sold to European citizens through these products is not improving to an extent to meet public health objectives.

## 1. Introduction

Noncommunicable diseases (NCDs) are responsible for the largest share of total health-related burden in the European Union (EU): they account for approximately 9 out of every 10 deaths and disability adjusted life years (DALYs) [1]. The four major NCDs, cardiovascular disease, cancer, chronic respiratory disease and diabetes mellitus, are responsible for at least 25% of the total healthcare spending, which is equivalent to an economic cost of 2% of the EU gross domestic product [2]. Among the avoidable risk factors, energy-dense diets high in fat, sugars and salt and low in fibre are one of the key risk factors for NCDs, thus contributing to the high health and economic burden linked to them [3,4].

Many national programs and strategies, both voluntary and mandatory, are in place to regulate marketing of front-of-pack labelling of such products. In addition, efforts to improve the nutritional quality of the food offerings are underway at the EU level through voluntary policies (Frameworks for National Salt Initiatives (2008) [5], National Initiatives on selected nutrients (2011) [6], the Annexes on Saturated Fat (2012) [7] and Added Sugars (2015) [8] and the roadmap for Action on Food Product Improvement (2016) [9]), and mandatory targets, standards and restrictions on nutrient content of industrially processed foods (i.e., Directive for the prohibition of added sugars in fruit juices (2012) [10] and the Regulation on trans fats (2019) [11]). More recently, the importance of healthy diets is also highlighted by the Farm to Fork Strategy [12] and Europe’s Beating Cancer Plan [13]. Beyond promoting a common European approach towards salt, sugars and fat intake reductions, these initiatives aim at encouraging a participatory engagement with public authorities and the food-processing sector, holding them accountable for food reformulation progress. 

Independent of the type of action undertaken at national and regional levels, monitoring and surveillance are crucial to understand the progress towards achieving the reformulation initiatives’ targets [14,15]. The comparison between food and nutritional data across Europe is often hampered by the lack of standardisation and harmonisation between national food classifications, nomenclatures or sources of data [16]. In this sense, one of the objectives of the Joint Action on Implementation of Validated Best Practices in Nutrition (Best-ReMaP) [17] is to work towards developing a European Standardised Monitoring system for the reformulation of processed foods for countries without existing systems, based on already available tools and expertise. However, given that this initiative started at the end of 2020, results will take time to materialise. 

In the absence of an existing and consolidated system to monitor Europe-wide reformulation efforts, this paper aims to assess progress on the existing commitments across European countries between 2015 and 2018. For that, we combine detailed market share and sales data with nutrition composition data from Euromonitor International to calculate market-share weighted averages of salt, total sugars, saturated fat and fibre content in selected food groups—most of them prioritised in several European frameworks. By integrating changes in nutritional composition of the food offer with changes in sales, this approach also provides insights into the ‘true’ average nutrient contents sold to consumers through different packaged food and soft drinks categories. Despite not measuring exactly what individuals have consumed, market data can be used by researchers and policymakers to keep track of the overall nutritional quality of the packaged food and drinks offered to and purchased by citizens [18,19,20,21,22,23,24].

The limited availability of food supply and composition data poses several challenges on assessing reformulation initiatives, which may explain why empirical peer-reviewed studies in this area have been relatively sparse. Studies reporting de facto changes have mainly taken advantage of nationally representative cross-sectional surveys to analyse the nutritional quality of the food supply over time. Most of these studies have mainly focused on evaluating the trends of a specific nutrient in selected food groups [19,21,23,25,26], or intended to compare the nutritional quality of similar food products across different countries at a specific point in time [16,18]. Compared to national dietary surveys and food composition tables, a third-party can offer more granular, frequent and standardised information on food sales and composition data disaggregated by type of product and brands, also avoiding reliance on individual recall [24]. Therefore, through the use of detailed and up-to-date commercially supplied food sales and composition data for many European countries, we attempt to reduce the literature gap by assessing the nutritional quality of packaged food and soft drinks sold through the retail sector, looking at several nutrients and food groups. By directly comparing the recent trends among many European countries, our findings allow the identification of food groups and nutrients that have presented progress as well as those that require more attention from policymakers, industry and consumers. 

## 2. Materials and Methods

### 2.1. Data Source

Euromonitor International [27] is a commercial global market research company providing data on the Packaged Food and Soft Drinks industries. Their Packaged Food and Soft Drinks databases include market-sized data of food/drink subcategories in value and volume terms, in addition to product/brand and company shares—which are available in volume and value terms for Soft Drinks, but only in value for the Packaged Food industry. In addition, the Nutrition database displays nutrient market sales volumes, and brand volumes on retails sales as well as back-of-pack labelling information on nutrient content (energy, carbohydrates, sugars, fibre, fat, saturated fat, protein and salt) per 100 g (or 100 mL) for the Soft Drinks and Packaged Food industries. Packaged Food/Soft Drinks and Nutrition data have been available since 2005 and 2014 respectively, until 2019, covering 5 subcategories of Soft Drinks and 16 subcategories of Packaged Food. Both datasets are frequently updated with five years of back-trended historic data and five years of forecasts. 

Euromonitor employs a research methodology of sourcing nutrition data predominantly online through brand/retailer websites, with store checks carried out when the information cannot be found online. The Nutrition database only tracks products sold through retail outlets (excluding, for example, restaurants, cafés/bars and vending machines). Therefore, only retail sales are considered in this analysis. The company relies on: (i) the regular updates of the relevant retailer’s websites, and (ii) the accuracy of the data provided on the label—so it is assumed that manufacturers provide accurate and up-to-date information in line with EU regulations.

Within Europe, the company researches nutrition data for 19 of the EU27 countries (Belgium, Bulgaria, Czech Republic, Denmark, Germany, Ireland, Greece, Spain, France, Italy, Hungary, Netherlands, Austria, Poland, Portugal, Romania, Slovakia, Finland and Sweden), the United Kingdom and 2 countries that are part of the European Free Trade Association (EFTA), Norway and Switzerland. Market data is available for 28 European countries. As for all countries for which nutrition data is available market data also is, our study focuses on the 22 European countries listed above.

### 2.2. Data Collection and Processing

As there is no pre-existing integration of Packaged Food/Soft Drinks and Nutrition databases available in the company’s platform, we created a key variable with the lowest level of product disaggregation (product/brand), which enabled the matching between both databases. For this analysis, we focused on 14 categories and 152 subcategories (see Table A1, Appendix A) of packaged foods and soft drinks that commonly represent major sources of added sugars, salt, saturated fat and fibre across different European countries. Moreover, these were also targeted in several national priorities and plans as well as in regional initiatives, such as the 2011 EU Framework for National Initiatives on Selected Nutrients [6]. 

After compiling and extracting market and nutrition data at the product/brand level, we removed those entries with: (i) missing market share, (ii) zero market share, (iii) missing/zero market value/volume and (iv) missing sugars, saturated fat, salt and fibre content data. For each remaining product/brand, we calculated the annual volume sold (in litres for Soft Drinks and tonnes for all other products) by multiplying the market share (in %) and the annual volume sold of the corresponding subcategory. Given that the market share data in volume terms is available only for Soft Drinks in Euromonitor, we used the value share to calculate total volume sold for all other categories, which may overestimate the volume sold of products that are more expensive per unit volume. We later estimated the market coverage for each category/subcategory based on the sum of total volumes of products in each category and the total volume of the category directly extracted from Euromonitor. In 2020, Euromonitor modified the 2019 Nutrition edition data structure from a product/brand level to a stock-keeping unit (or SKU) approach, which no longer allows the direct integration of market and nutrition data at the product/brand level. Thus, to ensure consistency, repeatability and reproducibility of our analysis, we used sales data from 2015 and 2018, which corresponds to data released by Euromonitor in 2016 and 2019. 

Table 1 presents the final list of packaged food and beverages categories assessed in this paper, and the number of products/brands contained in each category and the respective market coverage by country in 2018. Overall, more than 23,000 products/brands were analysed across 14 packaged food and soft drinks categories, covering, on average, 72% of the market for these categories. It is also possible to notice that some categories such as Dairy, Soft Drinks, Sauces, Dressings and Condiments (S, D & C), Confectionary and Sweet Biscuits, present many product variants. Data for 2015 covers approximately 22,400 products/brands that jointly represent 68% of the market (Table A2, Appendix A), which suggests that the total number of products by category and their respective market coverage remained relatively constant over the 2015–2018 period. 

Despite the high number of products/brands, some categories (such as Rice, Pasta and Noodles (R, P & N) and Baked Goods) present a relatively lower market coverage, while others contained few products that jointly represent a significant part of the total market in some countries (e.g., three products/brands represented 66% of the Greek market for soups), which justifies the focus of the analysis of the nutrient contents at the category level.

### 2.3. Estimation of the Market-Share Weighted Average for Selected Nutrients

To evaluate the broader nutrient profile of the food offer in Europe, we took into account the market dynamics of each product category/subcategory by adopting a market-share weighted average approach [18,19,21]. For this, we calculated the total annual retail volume (in grams/millilitres) of product/brand *i* (*V_i_*) and their respective category *V* ($V=\sum_{i}V_{i}$). If *N_i_* represents the salt, sugars, saturated fat or fibre content of each product/brand *i* (in grams per 100 g/100 mL) and *M_i_* indicates their corresponding market share (in % volume for Soft Drinks and in % value for all other categories), we can calculate the weighted mean nutrient content ($\bar{N}$) at the category level as follows:

(1) $$\bar{N}=\frac{\sum_{i}\left(M_{i}N_{i}\right)}{\sum_{i}M_{i}}=\frac{\sum_{i}(V_{i}N_{i})}{V}$$

Based on these estimates, we later calculated per capita nutrient sales values (g/pc/day) using mid-year population data for 2015 and 2018 from the European Health for all Database (HFA-DB) [28] and scaled these estimates according to the respective market coverage (as presented in Table 1) to represent 100% of the retail market. Therefore, while we are able to identify the contribution of specific products/brands to the overall quantity of nutrients sold within major packaged food and soft drinks categories in the EU, we followed [20,22] and assumed that the remainder of the market presents the same characteristics and evolution as our sample. This assumption minimizes data distortion given that many categories present a relatively high market coverage, and its magnitude remained stable across the analysed years [22]. Additionally, this assumption is founded on the fact that small and local companies represented by the remainder of the market are less likely to have the capacity for reformulation [20]. 

### 2.4. Statistical Analysis

Data are reported as weighted mean, arithmetic mean, standard deviation, median, minimum and maximum. Linear regressions were used to generate two-tailed, homoscedastic Student’s t-tests to identify if there was a significant change in the weighted and arithmetic mean nutrient content of packaged food and soft drinks categories between 2015 and 2018. All data were analysed using the STATA/SE version 15.1, and R.

## 3. Results

### 3.1. Evolution of Daily per Capita Sales of Total Sugars, Saturated Fat, Salt and Fibre (g per Capita per Day)

**Figure 1:**
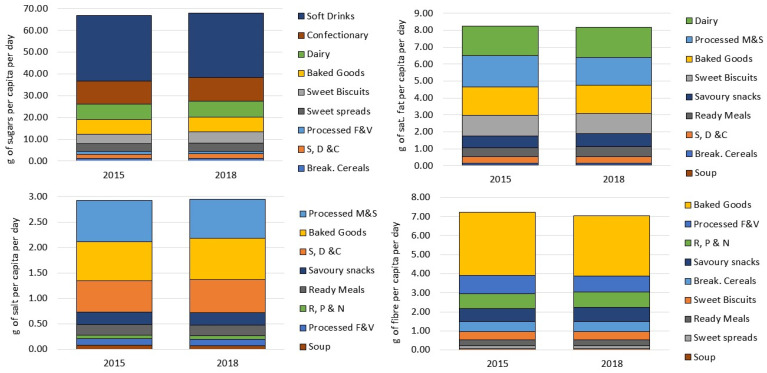
Sales of total sugars, saturated fat, salt and fibre (in g per capita per day) of packaged foods and soft drinks categories in Europe (2015–2018). Source: Elaborated by the authors based on data from Euromonitor (2020) and mid-year population estimates from the HFA-DB. Estimates were scaled by market coverage to represent 100% of the retail market.

Figure 1 presents our estimates of the daily per capita sales of total sugars, saturated fat, salt and fibre of all packaged food and soft drinks categories. These estimates were proportionally scaled by the respective market coverage (as presented in Table 1) to represent 100% of the retail market in Europe. For sugars, it is possible to observe that sales from Soft Drinks, Confectionary, Dairy and Baked Goods represent approximately 80% of per capita daily sugars sales across the 9 packaged food and soft drinks categories considered in this analysis in both years. Dairy, Processed M & S, Baked Goods and Sweet Biscuits account for the largest per capita daily sales of saturated fat. The largest volumes of salt per day were sold through Processed M & S, Baked Goods and S, D & C; for fibre, only one category (Baked Goods) accounted for approximately 45% of total per capita sales. The per capita amount of nutrients being sold every day through the analysed categories totalled almost 70 g for sugars, 8 g for saturated fat, 3 g for salt and 7 g for fibre. As a reference, the total amount of calories sold together with these nutrients corresponded to approximately 600, 622, 595 and 600 kcal/pc/day, respectively.

**Figure 2:**
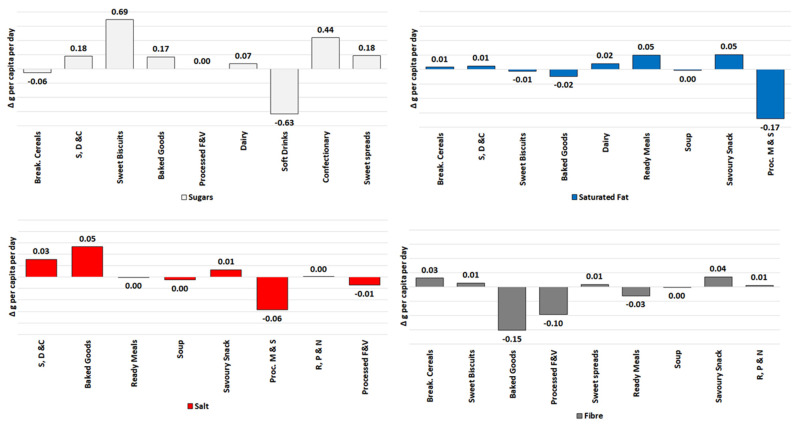
Variations in sales of total sugars, saturated fat, salt and fibre (in g per capita per day) of packaged foods and soft drinks categories in Europe (2015–2018). Source: Elaborated by the authors based on data from Euromonitor (2020) and mid-year population estimates from the HFA-DB. Estimates were scaled by market coverage to represent 100% of the retail market.

We also evaluated the absolute changes in daily per capita sales of total sugars, saturated fat, salt and fibre between 2015 and 2018 for all packaged food and soft drinks categories analysed in this paper (Figure 2). This can provide insights into the progress towards reducing sugars, saturated fat and salt as well as increasing fibre intakes in the population. Observed trends can be a result of innovation in the food offer, of changes in market share and volumes of products with certain nutrient profiles, or a combination of both. At the European level, we observed increases in sales of sugars for 6 out of the 9 categories analysed: Sweet Biscuits (0.69 g/pc/day), Confectionary (0.44 g/pc/day), S, D & C and Sweet Spreads (0.18 g/pc/day), Baked Goods (0.17 g/pc/day)and Dairy (0.07 g/pc/day). Soft Drinks and Breakfast Cereals presented a decrease of 0.63 and 0.06 g/pc/day, respectively. For saturated fat, a notable reduction was estimated for Processed M & S (−0.17 g/pc/day), and increases of 0.05 g/pc/day were observed for Ready Meals and Savoury Snacks, as well as for Dairy (0.02 g/pc/day). Breakfast Cereals and S, D & C presented variations of less than 0.01 g/pc/day. In addition, more salt was sold through Baked Goods (0.05 g/pc/day), S, D & C (0.03 g/pc/day) and Savoury Snacks (0.01 g/pc/day) between 2015 and 2018, while for Processed M & S and Processed F & V, we estimated reductions of 0.06 and 0.01 g/pc/day. Less fibre was also sold through Baked Goods (−0.15 g/pc/day), Processed F & V (−0.10 g/pc/day) and Ready Meals (−0.03 g/pc/day); conversely, more fibre was present in sales of Savoury Snacks (0.04 g/pc/day) and Breakfast Cereals (0.03 g/pc/day). 

We also found heterogeneous patterns when scrutinizing the changes in the amount of nutrients sold in packaged foods and soft drinks per country. By focusing only on the categories which presented the largest variations in the analysed period, Figure 3, Figure 4, Figure 5, Figure 6, Figure 7, Figure 8, Figure 9 and Figure 10 compare, in detail, the 2018 levels of daily per capita nutrients sold through selected categories with the respective estimated variation between 2015 and 2018 for all countries. Figure A1, Figure A2, Figure A3, Figure A4, Figure A5, Figure A6, Figure A7, Figure A8, Figure A9 and Figure A10 (Appendix A) present the results for other packaged food categories. 

**Figure 3:**
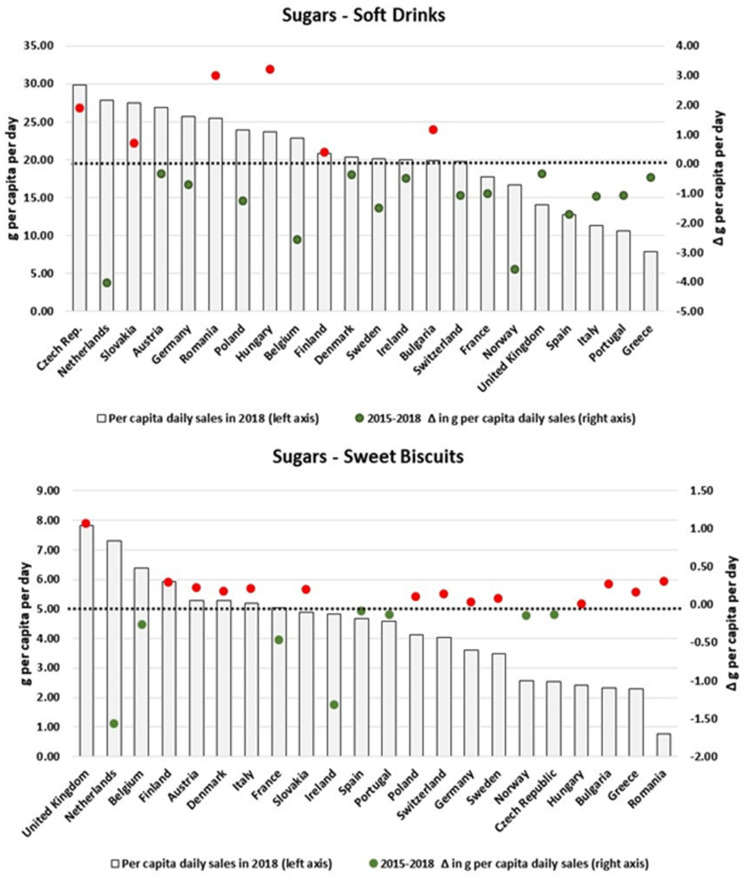
Estimates of total sugars sold through Soft Drinks and Sweet Biscuits in 2018 and the respective 2015–2018 variation (in g/pc/day). Source: Elaborated by the authors based on data from Euromonitor (2020) and mid-year population estimates from the HFA-DB. Estimates were scaled by market coverage to represent 100% of the retail market. The right-hand axis represents the nutrient variation in g per capita per day (green for negative values, red for positive values), and the dotted black line indicates the variation of 0 g per capita per day as a reference.

**Figure 4:**
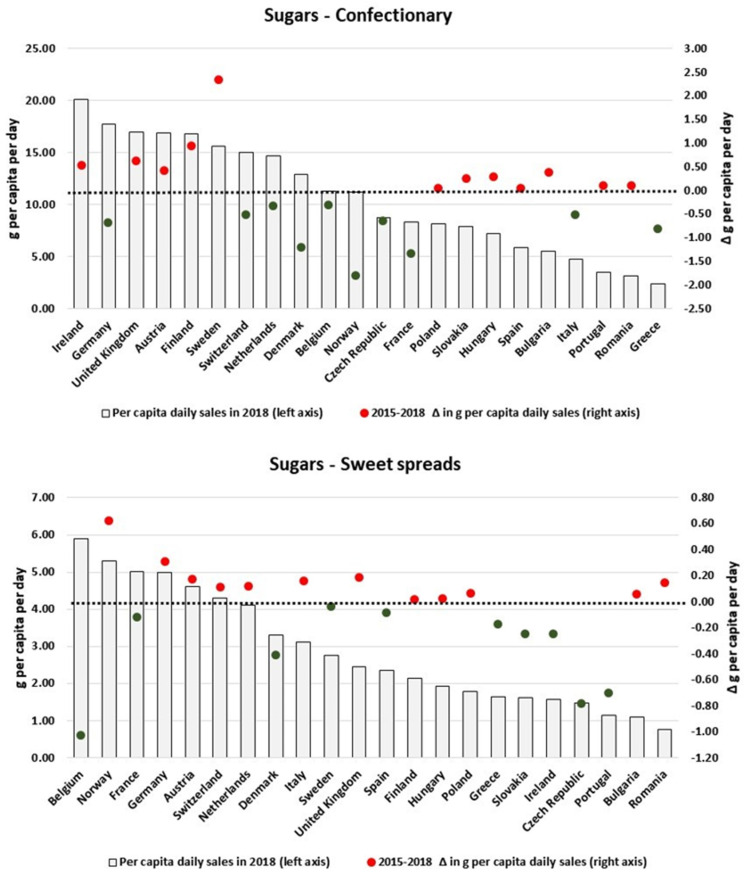
Estimates of total sugars sold through Confectionary and Sweet Spreads in 2018 and the respective 2015–2018 variation (in g/pc/day). Source: Elaborated by the authors based on data from Euromonitor (2020) and mid-year population estimates from the HFA-DB. Estimates were scaled by market coverage to represent 100% of the retail market. The right-hand axis represents the nutrient variation in g per capita per day (green for negative values, red for positive values), and the dotted black line indicates the variation of 0 g per capita per day as a reference.

For Soft Drinks, despite the regional decrease in the per capita sugars sales attributable to this product category, 6 countries (Hungary, Romania, Bulgaria, Czech Republic, Slovakia and Finland) had their respective sales increased between 2015 and 2018. In the case of Hungary and Bulgaria, this was equivalent to an additional more than 3 g/pc/day of sugars sold to consumers. Mixed results are also found for Sweet Biscuits: notably, while few countries with large per capita levels of total sugars sales (e.g., the Netherlands, Belgium, France and Ireland) had their sales reduced, countries with relatively lower sales of sugars (such as Romania, Greece and Bulgaria) increased their per capita sugar sales attributable to these products between 2015 and 2018. Conversely, for Confectionary, relatively large rises in daily per capita volumes of total sugars sales observed in Ireland, UK, Austria, Finland and Sweden might explain the overall increase at the European level. Similar patterns are also visualised for Sweet Spreads and the estimated increase in sugars sold as Sweet Spreads in Norway, Germany, Austria, Switzerland and the Netherlands. Figure 3 and Figure 4 summarize our estimates of total sugars sold through Soft Drinks, Sweet Biscuits, Confectionary and Sweet Spreads in the analysed period, in which red and green colours show respective increases and decreases in our daily per capita estimates. 

**Figure 5:**
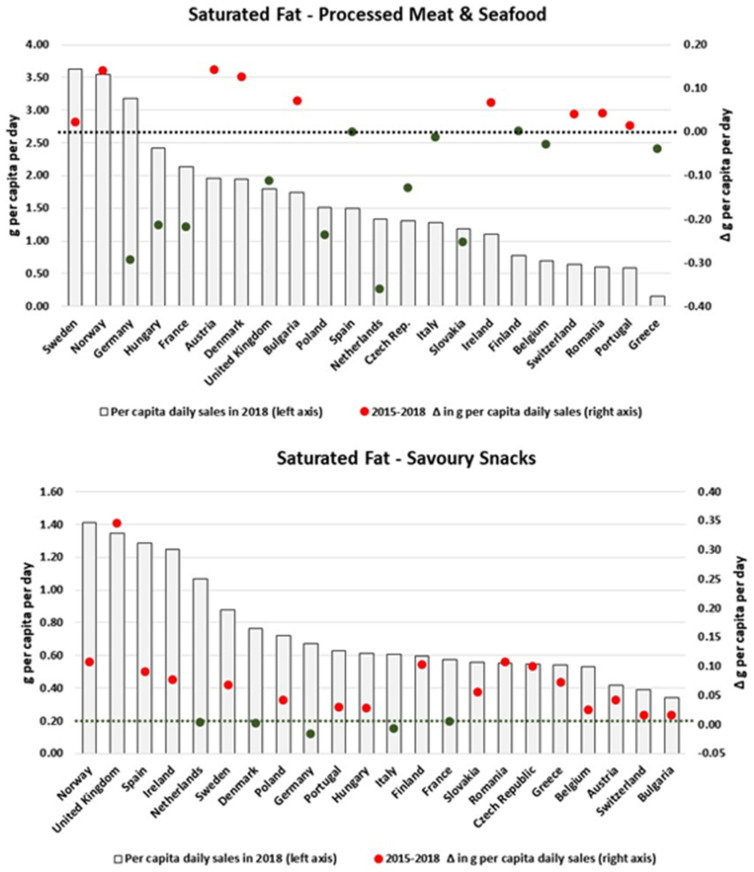
Estimates of saturated fat sold through Processed M & S and Savoury Snacks in 2018 and the respective 2015–2018 variation (in g/pc/day). Source: Elaborated by the authors based on data from Euromonitor (2020) and mid-year population estimates from the HFA-DB. Estimates were scaled by market coverage to represent 100% of the retail market. The right-hand axis represents the nutrient variation in g per capita per day (green for negative values, red for positive values), and the dotted black line indicates the variation of 0 g per capita per day as a reference.

**Figure 6:**
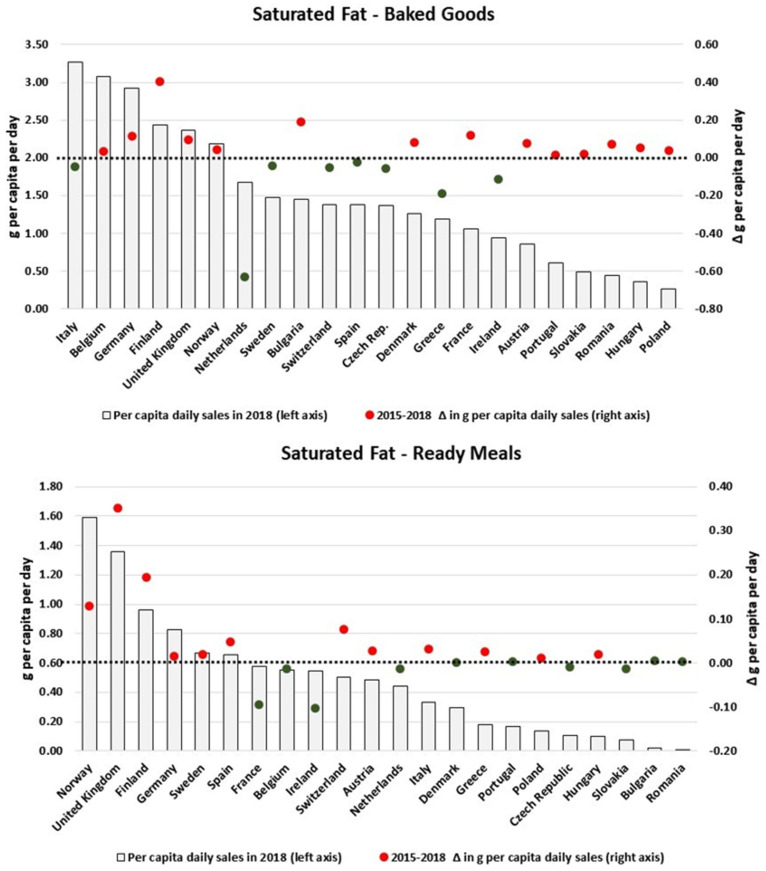
Estimates of saturated fat sold through Baked Goods and Ready Meals in 2018 and the respective 2015–2018 variation (in g/pc/day). Source: Elaborated by the authors based on data from Euromonitor (2020) and mid-year population estimates from the HFA-DB. Estimates were scaled by market coverage to represent 100% of the retail market. The right-hand axis represents the nutrient variation in g per capita per day (green for negative values, red for positive values), and the dotted black line indicates the variation of 0 g per capita per day as a reference.

Figure 5 and Figure 6 show that there is also a lot of variability in the amount of saturated fat sold through Processed M & S, Savoury Snacks, Ready Meals and Baked Goods across Europe. With relatively high levels of sales, Germany, Hungary and France presented reductions of approximately 0.3 g/pc/day on the respective daily per capita volumes of saturated fat sold as Processed M & S. However, with the exception of Germany, the Netherlands, Denmark, Italy and France, all countries presented an increase in the per capita levels of saturated fat sold as Savoury Snacks. Similar patterns were also observed for Baked Goods and Ready Meals. 

**Figure 7:**
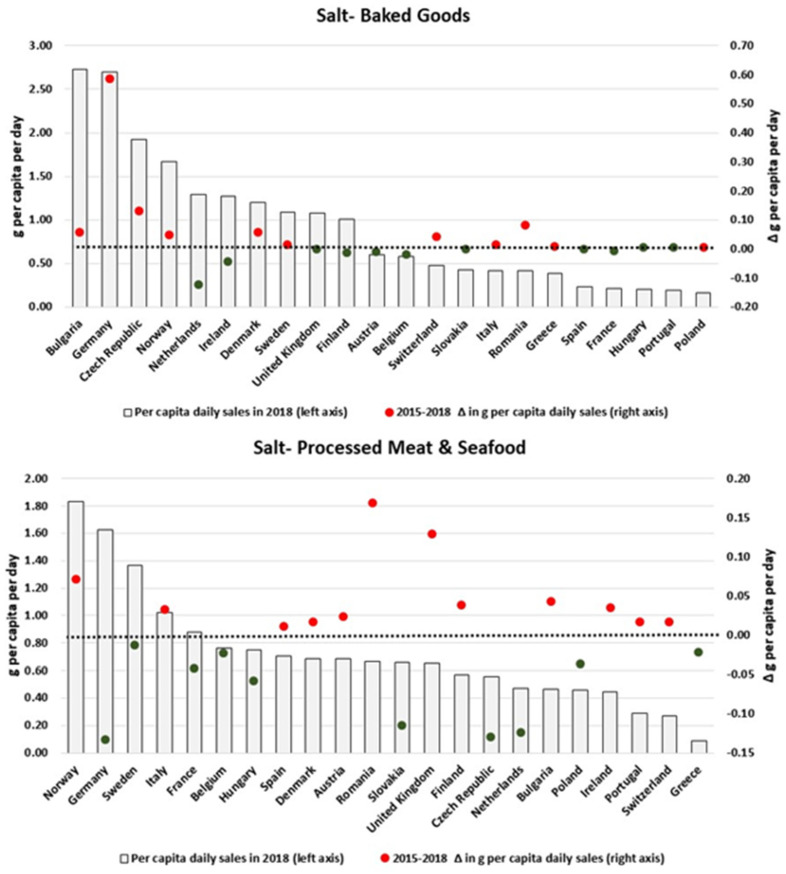
Estimates of salt sold through Baked Goods and Processed M & S in 2018 and the respective 2015–2018 variation (in g/pc/day). Source: Elaborated by the authors based on data from Euromonitor (2020) and mid-year population estimates from the HFA-DB. Estimates were scaled by market coverage to represent 100% of the retail market. The right-hand axis represents the nutrient variation in g per capita per day (green for negative values, red for positive values), and the dotted black line indicates the variation of 0 g per capita per day as a reference.

**Figure 8:**
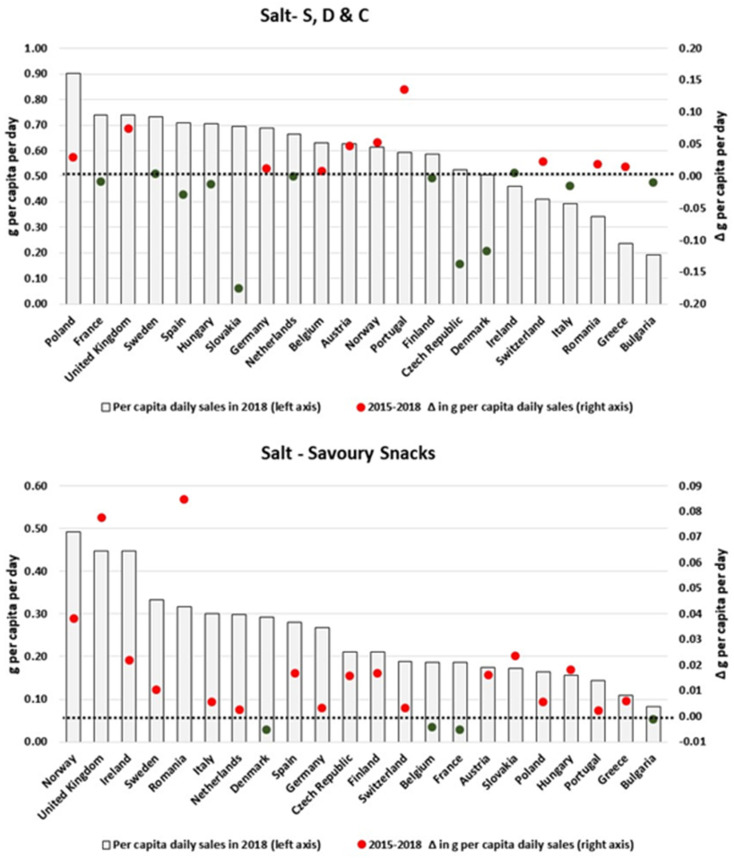
Estimates of salt sold through S, D & C and Savoury Snacks in 2018 and the respective 2015–2018 variation (in g/pc/day). Source: Elaborated by the authors based on data from Euromonitor (2020) and mid-year population estimates from the HFA-DB. Estimates were scaled by market coverage to represent 100% of the retail market. The right-hand axis represents the nutrient variation in g per capita per day (green for negative values, red for positive values), and the dotted black line indicates the variation of 0 g per capita per day as a reference.

As shown in Figure 7, daily per capita volumes of salt sold as Baked Goods have mainly increased in countries with relatively higher levels of sales (e.g., Germany, Czech Republic, Norway and Bulgaria). For Processed M & S (Figure 7) and S, D & C (Figure 8), results are more heterogeneous across countries: while notable decreases were observed for Germany, Slovakia, Czech Republic and the Netherlands for the former and Slovakia, Czech Republic and Denmark for the latter, proportional increases were also estimated for Romania and the UK, as well as the UK and Portugal, respectively. However, the per capita volumes of salt sold as Savoury Snacks appeared to have consistently increased in all countries, with the exception of Belgium, Denmark, France and Bulgaria.

**Figure 9:**
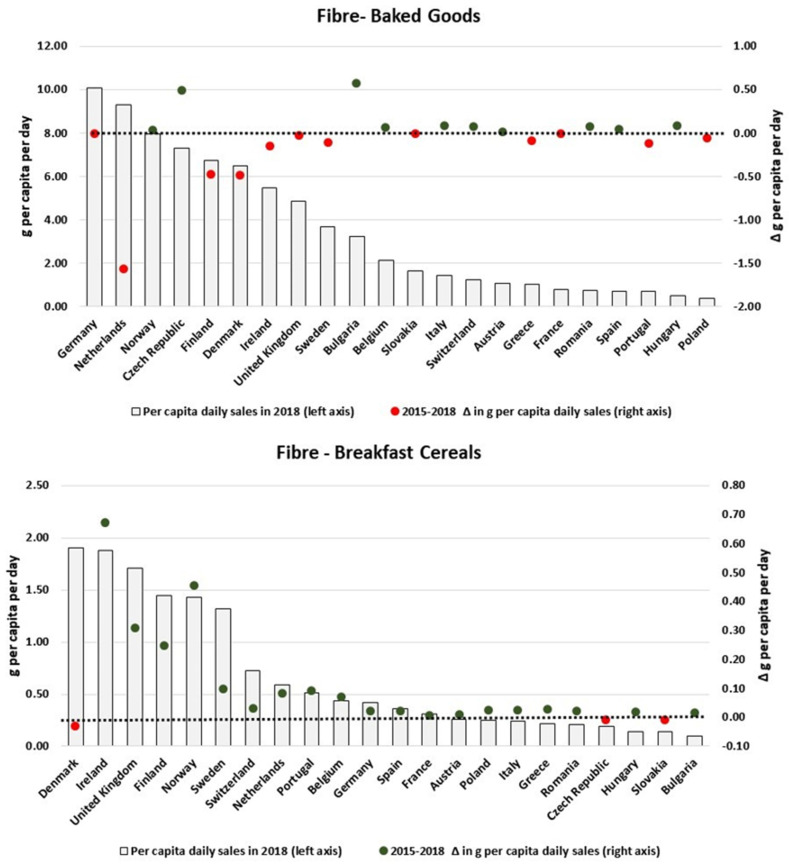
Estimates of fibre sold through Baked Goods and Breakfast Cereals in 2018 and the respective 2015–2018 variation (in g/pc/day). Source: Elaborated by the authors based on data from Euromonitor (2020) and mid-year population estimates from the HFA-DB. Estimates were scaled by market coverage to represent 100% of the retail market. The right-hand axis represents the nutrient variation in g per capita per day (green for positive values, red for negative values), and the dotted black line indicates the variation of 0 g per capita per day as a reference.

**Figure 10:**
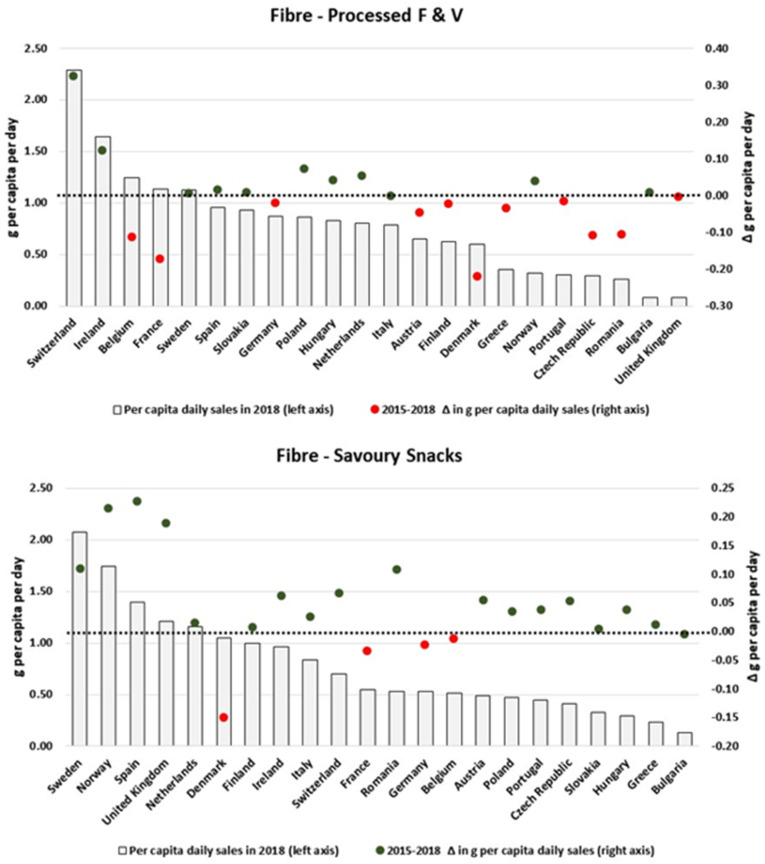
Estimates of fibre sold through Processed F & V and Savoury Snacks in 2018 and the respective 2015–2018 variation (in g/pc/day). Source: Elaborated by the authors based on data from Euromonitor (2020) and mid-year population estimates from the HFA-DB. Estimates were scaled by market coverage to represent 100% of the retail market. The right-hand axis represents the nutrient variation in g per capita per day (green for negative values, red for positive values), and the dotted black line indicates the variation of 0 g per capita per day as a reference.

Figure 9 examines the overall changes in daily per capita sales of fibre attributable to Baked Goods and Breakfast Cereals. For the first category, the reductions observed at the European level reflect the decreases estimated for countries with relatively high levels of fibre sales (e.g., the Netherlands, Denmark and Finland). Similarly, countries such as Ireland, the UK, Finland and Norway have presented increases of more than 0.2 g/pc/day in their respective per capita fibre sales of Breakfast Cereals; at the same time, almost no expressive variation was observed for countries with relatively low levels per capita of fibre sales coming from Breakfast Cereals. Conversely, more heterogeneous results were found for Processed F & V and Savoury Snacks (Figure 10).

### 3.2. Evolution of the Sugars, Saturated Fat, Salt and Fibre Content

#### 3.2.1. Sugars

Table 2 presents the overall results as distributions of total sugars in grams per 100 mL for Soft Drinks and grams per 100 g for all other packaged food categories (Breakfast Cereals, S, D & C, Sweet Biscuits, Baked Goods, Processed Fruits, Dairy, Soft Drinks, Confectionary and Sweet Spreads) across Europe in 2015 and 2018. Confectionary, Sweet Spreads and Sweet Biscuits are the categories with the highest levels of weighted mean sugars content. With the exception of Sweet Spreads, the sales-weighted mean sugars are lower than the arithmetic mean for all categories, which indicates that more sales came from the low-sugar products in 2015 and 2018. The statistics related to the extremes of the distribution (maximum and minimum) suggest that outliers—that is, products with highest and lowest sugars content—might have remained in the market, and the relatively high standard deviations across all categories disclose a large heterogeneity of nutrient profiles at the product/brand level. As an example, for Breakfast Cereals, there were specific types of Hot Cereals and Flakes with sugars content of 0.5 and 68 g/100 g, respectively. Similarly, under Confectionary, it is possible to identify medicated confectionery with sugar-free formulations and standard mints, with respective sugars content of 0 and 99.2 g/100 g. The differences between the arithmetic mean and median point to the fact that, with the exception of Breakfast Cereals, Confectionary and Sweet Spreads, all categories present more products with lower-than-the average sugars content.

Considering the change in total sugars content over time, the difference in means reports decreases in both market-weighted and arithmetic mean sugars content. For Breakfast Cereals, they are −4.4% (−0.7 g/100 g) and −2.5% (−0.4 g/100 g), respectively; for Soft Drinks, −3.8% (−0.3 g/100 mL) and −2.8%, (−0.3 g/100 mL), respectively; for Dairy, −1.2% (−0.1 g/100 g) and −0.3% (−0.04 g/100 g), respectively; for Sweet Spreads, −3.3% (−1.6 g/100 g) and −0.4% (−0.2 g/100 g), respectively. This denotes that, on average, the total sugars level content of the products offered was reduced from 2015 to 2018 and, in addition to this, there were proportionally less sales of products with sugars content higher than the arithmetic mean and more sales with content lower than it. Sweet Biscuits, Baked Goods, Processed Fruits and Vegetables (F & V) and S, D & C faced increases in the overall sugars content between 2015 and 2018 for both analysed means. However, for Confectionary, an estimated decrease was only observed for the weighted mean sugars content. However, the differences between weighted means were statistically significant only for Soft Drinks (*p* < 0.05), Sweet Biscuits (*p* < 0.05) and Breakfast Cereals (*p* < 0.10). Given that the arithmetic means of sugars content did not present a significant variation, these changes can be mainly explained by increases/decreases in sales volume of products with lower/higher sugar content, in the case of Soft Drinks and Breakfast Cereals, and increases/decreases in sales volume of products with higher/lower sugar content, particularly for Sweet Biscuits. These statistically significant variations are also aligned with the estimated variations in per capita nutrient sales, reported in the previous sections.

Table A3 (Appendix A) shows the variations (in g/100 and in %) of the market share weighted mean sugars content across countries. Mirroring the aggregated results at the European level, decreases in the weighted mean sugars content for Breakfast Cereals, Soft Drinks, Confectionary, Sweet Spreads and Dairy were observed for more than 70% of the countries—with notable exceptions of increases in Bulgaria (4% or 0.4 g/100 mL), Czech Republic (6% or 0.5 g/100 mL) and Portugal (16% or 1.3 g/100 mL) in the case of Soft Drinks, and Czech Republic (5% or 0.5 g/100 g), Greece (5% or 0.4 g/100 g), Portugal (6% or 0.6 g/100 g), Poland (3% or 0.3 g/100 g) and Spain (9% or 0.9 g/100 g) for Dairy products. Some reductions in the weighted mean of Processed F & V were also estimated for most of the countries, with ranges from −1.7 g/100 g (or −26%) for Romania to −0.1 g/100 g (or −2%) for Italy. Similarly, substantial differences in the weighted mean sugars content were found for S, D & C and Baked Goods. For Sweet Biscuits, the majority of the countries presented either an increase or have maintained the same levels of sugars in the products belonging to this category. Overall, our results indicate that the weighted mean sugars content estimated for different European countries was reduced, on average, in only 2 out of the 9 analysed categories. However, in some countries, such as Denmark, Ireland and the Netherlands, declines in the weighted sugars content can be extended to 8 out of the 9 analysed categories. Nonetheless, the magnitude of these changes was rather small throughout all categories analysed: as an example, the weighted mean sugars content in Soft Drinks and in Processed F & V decreased by more than 5% in only 6 countries; for Confectionary, this was identified only in 4 countries.

#### 3.2.2. Saturated Fat

The statistics presented in Table 3 show that Sweet Biscuits, Processed M & S and Savoury Snacks have consistently presented the highest values of weighted mean saturated fat content. Except for Processed M & S, the weighted mean saturated fat content is lower than the respective arithmetic means for all categories in both analysed years, pointing out that products high in saturated fat were responsible for a comparatively smaller proportion of total sales. The variability of data—represented by the standard deviation—remained virtually unchanged in the period, underpinning the large heterogeneity of saturated fat content in categories such as Sweet Biscuits, Savoury Snacks, Dairy and Baked Goods. Since the arithmetic mean is higher than the median for all categories, the largest part of the products belonging to the analysed categories presented saturated fat content higher than the average.

Reductions in the market-weighted and arithmetic means of saturated fat were observed for Breakfast Cereals, S, D & C, Sweet Biscuits, Processed M & S, Soup and Savoury Snacks, while opposite trends were found for Dairy and Baked Goods. However, the hypothesis tests for differences between means unveiled a statistically significant (*p* < 0.05) reduction in the market-weighted mean only for Sweet Biscuits (−8.3% or −0.7 g/100 g) and Processed M & S (−5.7%, or −0.3 g/100 g), trends also observed in the analysis of nutrient content in per capita terms. No category presented statistically significant changes in the arithmetic mean of saturated fat content between 2015 and 2018.

Results were also heterogeneous at the country level (Table A4, Appendix A). Particularly for Breakfast Cereals, there were more countries reporting an increase in the weighted mean of saturated fat rather than a decrease. A notable example was Denmark (increments of 0.3 g/100 g or 42%). Results are relatively better for S, D & C and Processed M & S, for which more than 70% of the countries presented some reduction in the market-weighted saturated fat content. For these categories, the average change was −5% across the assessed countries. A similar trend was also observed for Sweet Biscuits and Savoury Snacks, for which declines in the weighted saturated fat content were estimated for 70% of the countries. Considering Sweet Biscuits, notable reductions of the weighted mean of saturated fat content were observed in the UK (−2.7 g/100 g or −30%), Netherlands (−1.9 g/100 g or −20%), Ireland (−1.8 g/100 g or −20%) and Sweden (−1.6 g/100 g or −19%). Likewise, the weighted saturated fat content in Processed M & S products sold in the Netherlands, Poland and Slovakia decreased by more than 15% (or more than 0.7 g/100 g) over the last years. Similar trends were also identified for Savoury Snacks in Germany (−0.8 g/100 g or −15%).

#### 3.2.3. Salt

Table 4 presents the overall statistics for the distributions of salt content in eight food categories (Ready Meals, S, D & C, Baked Goods, Processed M & S, Processed F & V, R, P & N, Savoury Snacks, Soup) across European countries in 2015 and 2018. For Baked Goods, Processed M & S, Savoury Snacks and Processed F & V products, the market-weighted mean is higher than the arithmetic mean, which denotes that products with salt content higher than the arithmetic mean are proportionally and persistently sold in larger quantities than those with less. Furthermore, maximum values for S, D & C indicate that some of these products (a particular type of dry sauce) have several times more salt per 100 g than the daily salt intake recommended by the EU national health-related organisations.

The tests for differences between means presented a statistically significant decline in the market-weighted mean only for Processed M & S. However, none of the arithmetic means unveiled statistical evidence of reduced salt content for the selected category. This indicates that in this category, products with salt content less than the arithmetic mean gained market share over those with salt content above the arithmetic mean. Baked Goods was the only category to report a higher market-weighted mean in 2018 compared to 2015, in statistically significant terms (*p* < 0.0001). Its arithmetic mean also showed an increase in the respective saturated fat content, albeit not statistically significant. The upward/downward trend in sales of salt content of Baked Goods and Processed M & S can be also visualised in the estimated per capita daily changes.

There were substantial variations between 2015 and 2018 in salt levels of manufactured foods throughout Europe (Table A5, Appendix A). While more than 80% of the countries had some decrease in their overall weighted mean salt content sold as Ready Meals, Savoury Snacks and Processed F & V, for other categories, such as R, P & N, this was noted only for 40% of the countries. Czech Republic and Ireland reduced, by 0.3 g/100 g (−23%) and 0.2 g/100 g (−24%) respectively, the levels of salt sold through Ready Meals in 2018. Against 2015 levels, our estimates also suggest that the overall weighted mean salt content in S, D & C products sold in Czech Republic, Denmark and Slovakia reduced by more than 20%. At the same time, Greece and Portugal reported an increase in their weighted mean salt content of 13% (0.1 g/100 g) and 8% (0.2 g/100 g), respectively. The overall reductions observed for the Soup category are a reflection of the reductions of 2.7 g/100 g (or 41%) and 1.2 g/100 g (or 32%) in Poland and Portugal, respectively. For Baked Goods, increases in the weighted mean salt content were estimated for 40% of the analysed countries. Notable increases were found for Germany (0.3 g/100 g, or 27%) and Switzerland (0.1 g/100 g, or 7%). For Ready Meals, the reductions at the European level were mainly driven by the decreases observed in countries such as the Netherlands (−0.3 g/100 g, or −16%), Poland (−0.2 g/100 g, or −11%), the UK (−0.1 g/100 g, or −6%) and Germany (−0.1 g/100 g, or −5%).

#### 3.2.4. Fibre

Results for the overall changes in fibre content are present in Table 5. Since the market-weighted mean is higher than the arithmetic mean only for Baked Goods, R, P & N and Processed F & V, it is possible to note that products with relatively higher fibre content were proportionally and persistently sold in larger quantities than those with less fibre content. A significant decrease from 2015 to 2018 in the weighted mean estimates points out that there were proportionally less sales coming from products with higher than arithmetic mean fibre contents for Ready Meals. It is worth mentioning that the levels of fibre sold through products belonging to this category were already lower when compared to other analysed categories.

When assessing the evolution of the weighted mean fibre content at the national level, the results of Table A6 (Appendix A) also show remarkable heterogeneity among European countries. While approximately 60% of the countries had increased weighted mean fibre content in Sweet Spreads and Soups, the magnitude of this increment is, on average, not more than 2%. For other categories—such as Breakfast Cereals, R, P & N, Processed F & V and Savoury Snacks, only 30% of the countries presented an increase in their weighted mean fibre content over the past years. However, increases in the weighted mean fibre content were found for 6 or more categories in Switzerland, Bulgaria, Slovakia and Spain. Specifically, for Ready Meals, there were reductions observed in 9 out of the 24 analysed countries, reaching −0.6 g/100 g (−7%) in the case of Denmark and −0.3 g/100 g (−5%) in the Czech Republic.

#### 3.2.5. Paired vs. Unpaired Products/Brands

**Figure 11:**
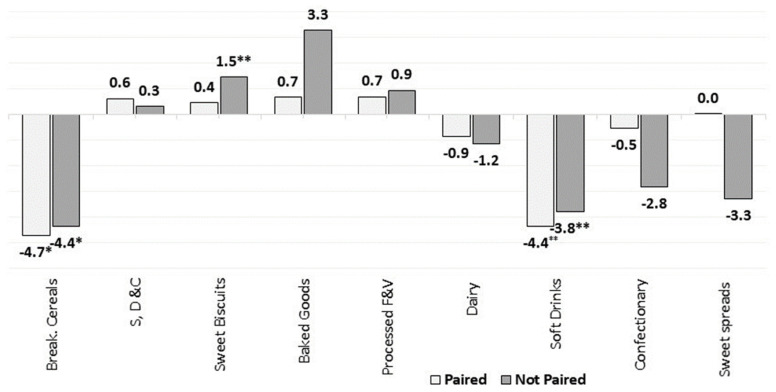
Percentage changes in the market-weighted mean sugars content in paired and unpaired packaged food and soft drinks products in Europe (2015–2018). Source: Elaborated by the authors based on data from Euromonitor (2020). Note: Percentage of products with available information both in 2015 and 2018: 98% for Breakfast Cereals, 88% for S, D & C, Sweet Spreads and Confectionary, 83% for Sweet Biscuits, 91% for Baked Goods, 82% for Processed Fruits and 94% for Dairy and Soft Drinks. Significance levels: * 10%, ** 5%.

**Figure 12:**
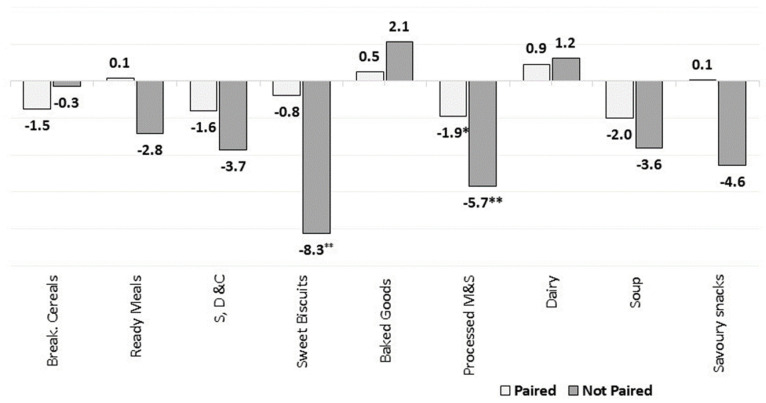
Percentage changes (in %) in the market-weighted mean saturated fat content in selected packaged food and soft drinks categories in Europe (2015–2018). Source: Elaborated by the authors based on data from Euromonitor (2020). Note: Percentage of products with available information both in 2015 and 2018: 98% for Breakfast Cereals, 88% for S, D & C, Savoury Snacks, Soup and Ready Meals, 83% for Sweet Biscuits, 91% for Baked Goods and Processed Meats, and 94% for Dairy. Significance levels: * 10%, ** 5%.

**Figure 13:**
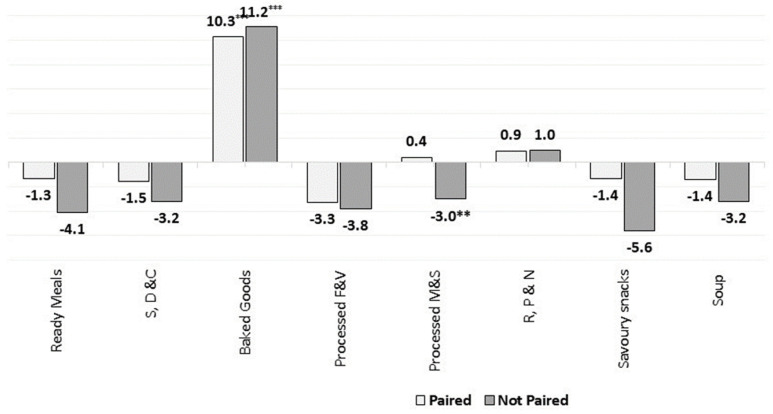
Percentage changes in the market-weighted mean salt content in paired and unpaired packaged food and soft drinks products in Europe (2015–2018). Source: Elaborated by the authors based on data from Euromonitor (2020). Note: Percentage of products with available information both in 2015 and 2018: 88% for S, D & C, Savoury Snacks, Soup and Ready Meals, 91% for Baked Goods and Processed Meats, and 84% for R, P & N. Significance levels: ** 5%, *** 1%.

**Figure 14:**
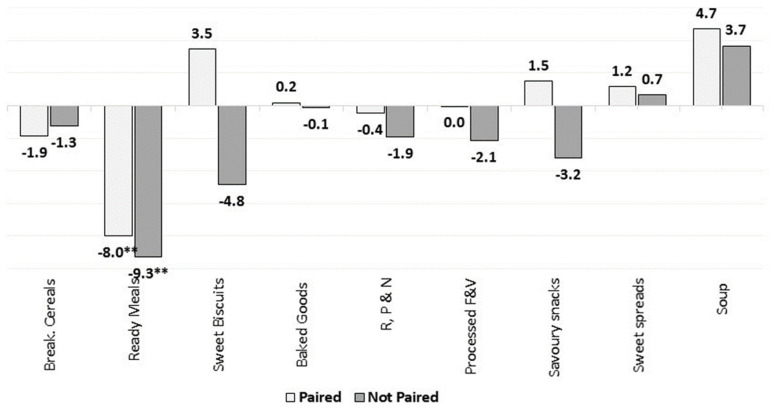
Percentage changes in the market-weighted mean fibre content in paired and unpaired packaged food and soft drinks products in Europe (2015–2018). Source: Elaborated by the authors based on data from Euromonitor (2020). Note: Percentage of products with available information both in 2015 and 2018: 98% for Breakfast Cereals, 88% for Savoury Snacks, Sweet spreads, Soup and Ready Meals, 91% for Baked Goods and Processed Meats, 82% for Processed Fruits, 84% for R, P & N and 83% for Sweet Biscuits. Significance levels: ** 5%.

We assessed the changes in the market-weighted nutrient mean only for products/brands that were available on the market both in 2015 and 2018. This enabled us to better analyse the overall market dynamics as well as the reformulation trends in Europe. Figure 11, Figure 12, Figure 13 and Figure 14 present the estimated changes in the market-weighted mean of sugars, saturated fat, salt and fibre content for products of the same brand and name observed in both years (“paired” products), which are plotted against the “unpaired” market-weighted nutrient means (reported in the Section 3.2.1, Section 3.2.2, Section 3.2.3 and Section 3.2.4), calculated based on all products available in Euromonitor’s database in any specific year. Significance levels are also shown for the weighted means that are statistically different from zero.

On average, approximately 89% of the products/brands analysed in this paper were present in both years in the Euromonitor database. For certain categories, such as Breakfast Cereals, this percentage was even higher, reaching 98%. This might be explained by the company’s methodological approach, which heavily relies on sampling only the most representative variants of a product/brand within each country. Therefore, despite not capturing the changes in nutritional quality of the whole food supply, our analysis reflects the overall nutritional trends of major products/brands offered by main retailers in Europe.

Figure 11 shows that paired products of Sweet Biscuits did not present significant increases in the market-weighted mean sugars content when compared to all products available on the market (i.e., unpaired products) in the analysed years. This suggests that the net effect of the introduction of products with higher sugars content and the discontinuation of options slightly lower in sugars might have driven the increase in this category. This outcome is also noted when assessing the weighted saturated fat mean content of products belonging to Sweet Biscuits and Processed M & S categories (Figure 12), as well as for the weighted mean of salt for Processed M & S (Figure 13).

In contrast, the weighted mean of sugars content in Breakfast Cereals and Soft Drinks dropped slightly more in paired products. This could indicate that products with higher market share had their sugars content reduced, and/or that products with a relatively low amount of total sugars per 100 g had their sales increased. It might also represent some reformulation effort from the leading brands towards reducing the sugars content in products of these categories. The weighted mean of salt content in paired products of Baked Goods also followed similar trends, as they had a smaller statistically significant increase when compared to unpaired products.

For fibre, Figure 14 shows identical trends in the market-weighted mean fibre content for paired and unpaired products in Ready Meals. This indicates that products with high fibre content may have been removed from the market, or products that have been introduced contained relatively low amounts of fibre. At the same time, it is also possible that some reformulation towards increasing the fibre content in some of those products persisting in the market was carried out.

## 4. Discussion

This paper analysed the trends of the nutritional quality of packaged food and soft drinks with respect to nutrients of public health priority, i.e., sugars, salt, saturated fat and fibre. We paired commercially available food composition data with market share and sales data and assessed 14 packaged foods and drinks categories sold in 22 European countries between 2015 and 2018. We provided novel insights on (i) how much of these nutrients were sold through these product groups in 2018 vs. 2015, (ii) how much the content of these nutrients changed in the food and drink offers and (iii) by analysing market-weighted mean nutrient contents, how the combination of nutrient content, sales and consumer preferences have evolved against public health nutrition objectives towards reducing intakes of sugars, saturated fat, salt and increasing those of fibre.

First, our daily per capita estimates for Europe indicate that the largest volumes of nutrient sales come from few product categories: Soft Drinks, Confectionary, Dairy and Baked Goods represent almost 80% of daily per capita sugars sales. For saturated fat, Dairy, Processed M & S, Baked Goods and Sweet Biscuits also accounted for a similar share of the estimated daily per capita sales. Salt sold through Processed M & S, Baked Goods and S, D & C accounted for 75% of the total per capita sales. At the same time, only two categories (Baked Goods and Processed F & V) were responsible for almost 60% of per capita sales of fibre. In addition, the results of the absolute changes in daily per capita sales in Europe suggest that few categories presented improvements in their nutritional content: sugars in Breakfast Cereals and Soft Drinks, saturated fat in Processed M & S, Sweet Biscuits and Baked Goods, salt in Processed M & S and Processed F & V and fibre in Savoury Snacks, Breakfast Cereals and Sweet Biscuits. Despite the progress observed in these categories, the magnitudes of the estimated changes lag behind when compared to the targets/benchmarks established by several initiatives, such as the 10% reduction for added sugars over a 5-year period [8], and the decreases of 16% and 5% for salt [5] and saturated fat [7] respectively, in 4-year periods. While the European aggregate estimates are useful to track changes in nutrient sales over time, they also hide severe heterogeneities among countries. For example, approximately 75% of the analysed countries presented some decrease in per capita levels of sugars sales attributable to Soft Drinks. On the other hand, the overall reduction in per capita sales of salt attributable to Processed M & S could be mainly attributed to only 4 countries. Likewise, few countries were the main contributors to the recent changes observed in daily per capita volumes of total sugars and fibres sold as Breakfast Cereals. Yet, despite some improvements observed in a few categories and nutrients, the progress against public health objectives on the amounts of sugars, saturated fat, salt and fibre being sold to European citizens through packaged food and soft drinks is modest. The amounts sold remain close to the 2015 levels (70 g/pc/day for sugars, 8 g/pc/day for saturated fat, 3 g/pc/day for salt and 7 g/pc/day for fibre on a basis of approximately 600 kcal/pc/day), and when compared to reference intake values from the Food Information to Consumers (FIC) regulation (90 g/pc/day for sugars, 20 g/pc/day for saturated fat, 6 g/pc/day for salt) and adequate intake of 25 g/pc/day for fibre [29], all on a reference basis of 2000 kcal/pc/day, it is possible to note that the levels of sugars and salt are of the most concern.

Second, beyond identifying great variability of the nutritional composition within and among packaged food and soft drinks categories, our estimates of the market-share weighted mean nutrient values suggest that the aggregated amount of sugars sold to European citizens was reduced only in 2 out of the 9 analysed categories (Breakfast Cereals and Soft Drinks), and even increased in the case of Sweet Biscuits. For saturated fat, reductions were observed in 2 out of the 9 assessed categories (Sweet Biscuits and Processed M & S), and for salt, reductions were observed only for products belonging to Processed M & S, and a significant increase was even estimated for Baked Goods. In general, the magnitudes of the improvements were rather small since the overall amounts of sugars, saturated fat and salt sold through these categories decreased by 3.3%, 4.4% and 2.1%, respectively. Moreover, statistically significant decreases in the market-share weighted fibre content were estimated for 1 out of the 9 assessed categories (Ready Meals) and overall, amounts of fibre sold across all product groups decreased by 2.1% between 2015 and 2018. In addition, our comparative analysis between the market-share weighted nutrient mean of paired and unpaired products point to large differences in sales and reformulation dynamics. For some categories and nutrients (such as saturated fat in Processed M & S, saturated fat and sugars in Sweet Biscuits), the changes might be explained by the net effect of the introduction/discontinuation of products with lower/high nutrient content between 2015 and 2018. For others (e.g., sugars in Breakfast Cereals and Soft Drinks and salt in Baked Goods), overall changes could be attributable to reductions/increases in market share of products with relatively high/low nutrient content, but also to some reformulation of those products which have been persistently sold in the European market.

Nevertheless, for the nutrients and categories in which a statistically significant change in the market-weighted nutrient content was identified, similar trends were also observed for their daily per capita nutrient sales. These findings consistently reflect different aspects of the overall quality of the food: the nutrient composition of the food supply and the average availability, market success of food and drinks with better or not so good nutrient profiles and the preferences of European citizens towards these products. In addition, the fact that most of the categories did not present a statistically significant change in their market-weighted nutrient content may indicate that just reformulating products or launching new ones with better nutrient profiles does not suffice to markedly impact sales and population diets if consumer preference for such products does not increase contemporaneously. Arguably, policies need to also address in parallel the food environment, supporting actions on educating, informing and incentivising consumers towards healthier choices.

Third, our results also suggest modest progress in the nutritional content of the food supply across the European countries. For some categories, such as Dairy, Sweet Biscuits and Confectionary, decreases of more than 5% in the respective market-share weighted total sugars content (in g/100 g) were observed only for 5 out of the 22 countries analysed; for others (e.g., S, D & C), there were only 2 countries achieving this level of reduction. Considering the EU Added Sugar Annex [8] and its 10% added sugar reduction target by 2020 against the 2015 baseline level, our findings suggest that countries risk falling short on their commitments to reduce the content of sugars in packaged foods and soft drinks. Similarly, the number of countries that had their market-share weighted mean of saturated fat reduced by more than 5% varies from 4 (e.g., for Dairy and Breakfast Cereals) to 9 countries (e.g., for Processed M & S). Decreases of more than 5% in the market-share weighted mean of salt were more frequent for Ready Meals and Processed F & V (9 countries) and Processed M & S and Savoury Snacks (7 countries). In the case of fibre, increases of more than 5% in the market-share weighted mean for Sweet Spreads and Soup were found for 7 countries, while for Sweet Biscuits and Ready Meals, this threshold was achieved in 6 and 5 countries, respectively. Results are even more worrying for other categories (such as Breakfast Cereals, Processed F & V and Savoury Snacks), as they point to decreases in the market-share weighted mean of fibre for more than 70% of the analysed countries.

Direct comparisons between our study and previous ones are limited by different methodologies, timeframe and product categorisation. However, our country-level results are consistent with the recent trends and degree of nutrient variability reported by previous peer-reviewed studies using similar methodological approaches and timeframe. For sugars, the authors of [19] used an annual cross-sectional study using nutrient composition data for products available online to assess changes in sales of soft drinks in the UK from 2015 to 2018. They estimated a 30% decline in the per capita volume of sugars sold from soft drinks in the UK between 2015 and 2018 (equivalent to a reduction of 4.6 g per capita per day), which were translated into a statistically significant reduction of 1.5 g/100 mL of the sales-weighted mean sugars content of soft drinks. Using the same methodology, the same authors estimated a 13.3% decrease (95% confidence intervals (CI): −19.2% and −7.4%) in the sales-weighted mean sugar content for breakfast cereals in the same period [20], close to our point-estimate of −9.0%. Similar analyses were conducted by [25], and they found that energy drinks presented a statistically significant reduction of 10% in their total sugars content (from 10.6 (±3.2 g) to 9.5 (±3.3 g) g/100 mL) between 2015 and 2017 in the UK. Manufacturers of the sugar-reduced products had either only reduced sugars or had alternatively reduced sugars, replaced with non-caloric sweeteners without changing the product name, for example, by calling the product ‘light’ and so on. Despite the observed reduction, the sugars content of energy drinks was still at concerning levels by the end of the study period. Particularly for Soft Drinks in the UK, our estimates point to a 5% reduction (−0.3 g/100 mL) in the market-share weighted sugars mean and a 5% decrease (−0.3 g/pc/day) in the daily per capita sales between 2015 and 2018.

Focusing on yogurts, the authors of [26] detected a highly significant reduction of 14% in the median total sugars contents in 2019 compared to those in 2016 (from 11.9 (CI: 8.8, 13.6) to 10.4 (CI: 6.6, 13.0) g/100 g) based on online data—nutrient information, serving size, size of pack, claims on pack and ingredients—collected from major UK supermarkets. When scrutinising paired products, i.e., products that were present with the same brand name both in 2016 and 2019, only 1/3 of the 539 products surveyed had reduced sugars contents, with a smaller mean difference of −0.65 g/100 g (CI: −0.78, −0.52). This suggests that the overall median had dropped due to the discontinuity of products with relatively higher sugars content and/or the introduction of new products with relatively lower sugars content. These reported trends are aligned with the results presented in this study and others [20]. Taking Dairy as a reference category, we calculated decreases of −0.7 g/100 g (or −6%) in the market-share weighted sugars mean for the UK between 2015 and 2018.

For salt, using Dutch food composition data with monitoring reports and relevant labelling information collected from the largest supermarket chain, the authors of [30] found no statistically significant change in sodium levels of processed meat products between 2011 and 2015 in the Netherlands. However, according to the authors, a reversal in this trend was expected to occur in the upcoming years as per the 2014 agreement between the Dutch government and the private sector, in which a 10% mandatory reduction target for meat products to be reached before the end of 2020 was established. The authors of [30] also estimated a significant reduction in the saturated fat levels for only one subcategory of meat products (heated meat products): from approximately 10 g/100 g (±3.0 g) in 2014 to 8 g/100 g (±3.0 g) in 2015. Notably, a 5% target for saturated fat was also established by the Government and the private sector for these products. For the 2015–2018 period, we calculated decreases of 16% (or −0.3 g/100 g) and 19% (or −1.1 g/100 g) in the market-share weighted salt and saturated fat mean for Processed M & S in the Netherlands, which suggests that the establishment of mandatory targets might have been effective in reducing salt and saturated fat levels in meat products in the country.

The grey literature also supports and complements our findings and the ones from peer-reviewed studies. A monitoring report from Public Health England (PHE) highlighted that there has been progress in some, but not all, food categories between 2015 and 2019. Sustained progress in sales-weighted average sugars was observed for breakfast cereals (−13.3% or −2.2 g/100 g) and yogurt and fromage frais (−12.9% or −1.6 g/100 g); remarkably, sales-weighted average sugars in soft drinks subject to the levy decreased by 44% (or −1.7 g/100 mL), even though overall sales volume increased by 14%. In contrast, sugars levels in chocolate and sweet confectionery remained relatively unchanged, with variations of −0.4% (−0.2 g/100 g) and −0.1% (−0.04 g/100 g). Overall, the average sugars reduction across all monitored food categories (including the ones previously mentioned) stands at 3% from 2015 to 2019, falling extremely short of the targeted 20% reduction by 2020 [31]. PHE also reported a slow progress in the salt reduction programme, in which just over half (52%) of the salt reduction targets for product averages set in 2014 were met by 2017. Retailers’ own brands made more progress than manufacturers towards achieving these targets, meeting 73% of them compared with manufacturers, who met 37%. However, where targets on maximum salt contents were set, 83% of in-home products met these targets vis-à-vis 71% for out-of-home products [32]. Another study for the Netherlands, released by the Ministry of Health, Welfare and Sports, found that the salt content in bread in 2016 was, on average, 19% lower than in 2011. Certain savoury sauces, soups, tinned vegetables and pulses, and chips, had between 12% and 25% less salt for the same period. However, despite the improvements in salt content, the average saturated fat and sugars content in most food categories remained at the same levels over the past years [33].

The results of this study are relevant and serve to document and provide evidence to policymakers, industry and citizens on the evolution of sugars, saturated fat, salt and fibre content of packaged foods and soft drinks sold in Europe. The strength of the market-share weighted average approach lies in its ability to capture the true market influence of products that could otherwise be interpreted as outliers, such as those with a low nutrient content but high market share or, alternatively, those with a low market share but high nutrient content. It also gives greater impetus to the reformulation of leading market products and limits the impact of new and/or reformulated products with improved nutrient contents but low market share. It should also be noted that this approach does not only capture producers’ changes in the food offer, it also reflects the market success of products with better nutritional quality with consumers, which also depends on consumer attitude, preference and motivation (e.g., shift towards existing low-sugar or sugar-free products).

However, despite being an interesting and effective data source to monitor the progress of the food quality across different countries, there are limitations on the use of commercial data and on the results generated by them [24]. Given that Euromonitor’s methodological approach heavily relies on sampling only the most representative variants of a product/brand within each country, our results should be interpreted as reflections of the behaviour and efforts of the market-leading brands and products rather than the performance of the overall food supply. Furthermore, Euromonitor has innovated its data collection methodology using automatic scraping of product information from online stores, affecting the data in the nutrition module from 2019 onwards. Whilst this comes with advantages, such as nutrition information at the stock keeping unit (or SKU) level rather than most representative variant of brands, this break in methodology does not easily allow for assessing trends in daily per capita nutrient sales figures and alike from before 2019 and 2019 onwards. We are also constrained by the accuracy of the information presented on the label, as it is assumed that manufacturers provide accurate and up-to-date information, in line with EU regulations. Additionally, in the absence of market share in value terms in the Euromonitor database, the assumption made in this paper to use value shares as the equivalent to volume share can overestimate the market share of products that are more expensive per unit volume, as well as the point-estimates of the weighted average nutrient content. Lastly, as data on package size is not available in Euromonitor’s Nutrition database, it was not possible to separate the effects of reformulation and changes in package size on the market-share weighted nutrient mean estimates.

In this sense, the development of a standardised system to track wide reformulation initiatives across the EU Member States appears to be very much needed [14], and the outcomes of the joint endeavour of EU countries within the Joint Action Best ReMaP [17], which builds on previous pan-European efforts, is expected to reduce this gap. This system could ensure sustainability on the monitoring and surveillance of nutrition policies at the country and the European level, as well as to help in identifying best practices at a bigger scale, and to establish unbiased targets for reduction of sugars, salt and saturated fat, as well as increments in fibre among countries. Improvements in the overall quality of the food offer would also contribute to enhancing diets (a priority in several EU Strategies, such as the Farm to Fork Strategy or Europe’s Beating Cancer Plan), which could be then translated into significant benefits for the population by promoting health and reducing the health and economic burden of NCDs.

Future research is needed to better understand the supply and demand drivers behind changes in nutrition content, as well as the impact of integrated strategies on sales, purchases and ultimately diets and health. By combining food composition and purchasing data, the methodology developed in [34] and applied to the French context in [22] allows the decomposition of the overall change in the nutrient content into what can be attributable to: (i) consumers switching from some products to others, (ii) firms reformulating food products and (iii) net product introduction (the combination of new products and product withdrawals). Additional studies could also shed light on the evolution of portion sizes across different food categories and countries, as well as its impact on the overall market-share weighted mean of selected nutrients.

## 5. Conclusions

Using commercially available third-party data, this analysis shows some progress, albeit modest, in Europe towards the direction desired by public health proponents, namely the reductions in people’s salt, sugars and saturated fat intakes. We also demonstrated that there is little evidence of increases in fibre content from the packaged food offers covered in our study. Beyond providing a better understanding of recent trends of packaged food’s nutrient content to governments, industry sector and the research community, our results allow the identification of areas of the food supply most urgently requiring action, as well as highlight those that are making advances. In addition, our analyses indicate that for food reformulation strategies to be effective, products with better nutrient profiles need to compete well on the market. However, these findings are limited by the characteristics of the third-party data used in this study, as well as the assumptions related to the market shares. We suggest to further research the aspect of (lack of) market success, including consumer demand, and assess the effectiveness of demand-side policies, in particular in parallel with reformulation strategies, on sales or purchases and finally, population diet and health.

## Figures and Tables

**Table 1:** Number of products extracted from the Euromonitor database and their respective market coverage (in %) in 2018, per category and country.

**Country**	**Break. Cereals**		**Dairy**		**Soft Drinks**		**Ready Meals**		**S, D & C**		**Soups**		**Sweet Spreads**	
		**% Cov.**		**% Cov.**		**% Cov.**		**% Cov.**		**% Cov.**		**% Cov.**		**% Cov.**
Austria	25	72%	109	59%	154	76%	34	79%	84	83%	16	86%	24	73%
Belgium	48	63%	172	81%	125	65%	51	73%	123	78%	20	56%	28	92%
Bulgaria	35	57%	121	50%	136	78%	21	80%	112	87%	16	91%	25	35%
Czech Rep.	49	84%	194	84%	149	58%	31	67%	141	96%	19	88%	21	72%
Denmark	41	71%	122	86%	134	74%	32	85%	119	99%	15	84%	25	72%
Finland	40	78%	142	87%	186	79%	42	88%	114	92%	26	94%	16	60%
France	52	68%	167	69%	150	76%	51	70%	110	73%	19	83%	36	70%
Germany	42	69%	175	74%	152	64%	51	71%	106	82%	17	83%	22	62%
Greece	31	69%	121	68%	94	83%	42	87%	101	90%	3	66%	18	51%
Hungary	63	79%	236	80%	253	83%	33	71%	123	73%	4	58%	27	50%
Ireland	56	43%	136	67%	96	77%	34	57%	112	74%	18	79%	26	68%
Italy	34	74%	149	70%	125	70%	73	49%	128	87%	18	87%	27	76%
Netherlands	36	57%	157	80%	127	59%	67	76%	177	84%	23	88%	33	70%
Norway	50	75%	112	92%	119	88%	25	72%	102	87%	14	91%	30	88%
Poland	59	83%	171	69%	166	80%	49	79%	128	84%	29	100%	31	69%
Portugal	43	64%	97	73%	89	45%	32	67%	87	100%	12	100%	21	82%
Romania	39	52%	155	78%	98	68%	21	52%	121	75%	7	69%	22	64%
Slovakia	39	85%	147	80%	183	75%	32	89%	114	91%	14	70%	19	52%
Spain	33	67%	144	71%	128	88%	48	54%	127	68%	27	78%	24	61%
Sweden	63	81%	145	82%	174	79%	47	88%	174	91%	26	92%	28	67%
Switzerland	41	70%	149	78%	112	81%	25	64%	65	86%	15	91%	12	57%
UK	57	60%	153	75%	159	84%	62	75%	108	66%	37	79%	27	69%
Total (av.%)	976	69%	3274	75%	3109	74%	903	72%	2576	84%	395	82%	542	66%
**Country**	**Confectionary**		**Savoury Snacks**		**Sweet Bisc.**		**Baked Goods**		**Proc. M & S**		**R, P%N**		**Proc. F & V**	
		**% Cov.**		**% Cov.**		**% Cov.**		**% Cov.**		**% Cov.**		**% Cov.**		**% Cov.**
Austria	122	78%	65	78%	69	64%	31	44%	63	60%	14	55%	57	81%
Belgium	153	69%	67	80%	103	73%	58	49%	88	70%	25	52%	49	81%
Bulgaria	150	80%	91	85%	140	83%	57	46%	63	85%	15	34%	62	81%
Czech Rep.	147	78%	94	86%	95	79%	51	43%	67	82%	31	41%	63	100%
Denmark	139	83%	59	93%	63	80%	43	37%	93	96%	18	57%	61	100%
Finland	146	71%	72	82%	69	78%	52	55%	82	94%	23	48%	58	96%
France	130	55%	83	81%	103	83%	58	61%	104	65%	25	47%	53	82%
Germany	173	70%	69	88%	85	60%	39	55%	77	67%	24	78%	38	74%
Greece	91	78%	52	77%	63	79%	58	67%	61	88%	17	70%	40	79%
Hungary	143	62%	81	67%	102	68%	83	70%	100	77%	37	46%	60	67%
Ireland	132	68%	88	74%	82	96%	55	44%	33	67%	17	35%	20	61%
Italy	147	77%	93	67%	105	82%	91	42%	150	68%	44	64%	99	73%
Netherlands	158	74%	68	73%	75	68%	32	54%	77	94%	14	24%	50	80%
Norway	106	85%	62	84%	50	81%	48	62%	59	85%	13	34%	37	82%
Poland	157	80%	95	87%	111	81%	51	71%	83	99%	36	55%	73	87%
Portugal	91	68%	56	84%	71	74%	38	64%	63	78%	23	36%	37	76%
Romania	124	72%	71	77%	118	76%	43	61%	88	94%	19	22%	65	90%
Slovakia	123	71%	82	74%	100	78%	54	51%	53	92%	23	35%	42	78%
Spain	164	73%	72	74%	95	60%	49	69%	88	62%	26	39%	46	74%
Sweden	127	66%	100	83%	79	82%	61	58%	139	90%	33	37%	78	91%
Switzerland	120	56%	65	84%	65	55%	24	71%	33	78%	17	67%	25	87%
UK	177	63%	113	82%	106	88%	82	58%	81	82%	25	27%	57	73%
Total (av.%)	3020	72%	1698	80%	1949	76%	1158	56%	1745	81%	519	46%	1170	82%

Source: Elaborated by the authors based on data from Euromonitor (2020). Note: % Cov. = coverage (in %), S, D & C = Sauces, Dressings and Condiments; Proc. M & S = Processed Meat and Seafood; R, P & N = Rice, Pasta and Noodles; Proc. F & V = Processed Fruits and Vegetables.

**Table 2:** Summary statistics and variations of total sugars content (in g/100 g or mL) for selected packaged food and soft drinks categories in Europe.

**Category**	**2015**						**2018**					
	**Weighted Mean**	**Mean**	**SD**	**Median**	**Min**	**Max**	**Weighted Mean**	**Mean**	**SD**	**Median**	**Min**	**Max**
Break. Cereals	16.0	17.5	10.9	18.6	0.5	68.0	15.3	17.1	10.7	18.0	0.5	68.0
S, D & C	8.5	10.0	10.8	6.1	0.0	81.0	8.6	10.0	10.8	6.0	0.0	81.0
Sweet Biscuits	30.2	32.5	13.2	32.0	0.1	78.6	30.6	32.6	13.4	32.0	0.1	78.6
Baked Goods	9.3	15.4	15.6	8.0	0.5	86.8	9.6	15.5	15.6	8.6	0.5	86.8
Processed F & V	3.4	4.4	5.5	3.0	0.0	60.0	3.4	4.5	5.5	3.0	0.0	60.0
Dairy	10.6	12.8	8.1	12.1	0.0	59.0	10.5	12.8	8.1	12.0	0.0	59.0
Soft Drinks	8.1	9.8	14.5	9.0	0.0	95.0	7.8	9.5	14.1	8.9	0.0	93.3
Confectionary	52.4	53.4	18.5	54.0	0.0	99.2	50.9	53.5	18.6	54.0	0.0	99.2
Sweet spreads	48.2	42.5	18.6	51.0	0.7	67.9	46.6	42.3	19.0	50.9	0.7	67.9
**Category**	**Mean Difference (2015–2018)**											
	**Weighted**						**Arithmetic**					
	**g/100 g**		**%**		***p* Value**		**g/100 g**		**%**		***p* Value**	
Break. Cereals	−0.70		−4.4%		0.097		−0.44		−2.5%		0.375	
S, D & C	0.03		0.3%		0.320		0.00		0.0%		0.999	
Sweet Biscuits	0.44		1.5%		0.026		0.12		0.4%		0.783	
Baked Goods	0.30		3.3%		0.688		0.06		0.4%		0.934	
Processed F & V	0.03		0.9%		0.562		0.07		1.6%		0.770	
Dairy	−0.12		−1.2%		0.545		−0.04		−0.3%		0.862	
Soft Drinks	−0.31		−3.8%		0.020		−0.28		−2.8%		0.429	
Confectionary	−1.48		−2.8%		0.712		0.09		0.2%		0.870	
Sweet spreads	−1.58		−3.3%		0.708		−0.16		−0.4%		0.897	

Source: Elaborated by the authors based on data from Euromonitor (2020). Note: S, D & C = Sauces, Dressings and Condiments; Processed F & V = Processed Fruits and Vegetables. SD = standard deviation.

**Table 3:** Summary statistics and variations of saturated fat content (in g/100 g) for selected packaged food categories in Europe.

**Category**	**2015**						**2018**					
	**Weighted Mean**	**Mean**	**SD**	**Median**	**Min**	**Max**	**Weighted Mean**	**Mean**	**SD**	**Median**	**Min**	**Max**
Break. Cereals	1.44	1.68	1.95	1.00	0.00	12.00	1.44	1.66	1.89	1.00	0.00	12.00
Ready Meals	2.52	2.53	1.96	2.30	0.00	13.50	2.45	2.56	1.97	2.30	0.00	13.50
S, D & C	1.65	2.01	3.81	1.00	0.00	37.80	1.59	1.98	3.61	1.00	0.00	37.80
Sweet Biscuits	8.40	8.72	6.25	8.30	0.00	35.00	7.71	8.64	6.20	8.10	0.00	35.00
Baked Goods	2.29	4.26	4.77	2.30	0.00	23.00	2.34	4.26	4.80	2.30	0.00	30.00
Processed M & S	4.81	3.67	3.85	2.30	0.00	23.60	4.53	3.66	3.82	2.30	0.00	23.00
Dairy	2.49	3.96	5.06	2.20	0.00	40.00	2.52	3.99	5.03	2.20	0.00	40.00
Soup	0.78	1.57	2.77	0.70	0.00	20.00	0.75	1.55	2.71	0.70	0.00	20.00
Savoury snacks	4.62	5.27	5.10	3.40	0.00	47.70	4.41	5.18	5.06	3.30	0.00	47.70
**Category**	**Mean Difference (2015–2018)**											
	**Weighted**						**Arithmetic**					
	**g/100 g**		**%**		***p* Value**		**g/100 g**		**%**		***p* Value**	
Break. Cereals	0.00		−0.3%		0.880		−0.03		−1.6%		0.758	
Ready Meals	−0.07		−2.8%		0.936		0.03		1.1%		0.764	
S, D & C	−0.06		−3.7%		0.729		−0.03		−1.5%		0.769	
Sweet Biscuits	−0.69		−8.3%		0.045		−0.09		−1.0%		0.679	
Baked Goods	0.05		2.1%		0.870		0.00		0.0%		0.994	
Processed M & S	−0.27		−5.7%		0.049		−0.01		−0.2%		0.960	
Dairy	0.03		1.2%		0.639		0.02		0.6%		0.853	
Soup	−0.03		−3.6%		0.894		−0.03		−1.9%		0.880	
Savoury snacks	−0.21		−4.6%		0.754		−0.09		−1.8%		0.600	

Source: Elaborated by the authors based on data from Euromonitor (2020). Note: S, D & C = Sauces, Dressings and Condiments. SD = standard deviation.

**Table 4:** Summary statistics and variations of salt content (in g/100 g) for selected packaged food categories in Europe.

**Category**	**2015**						**2018**					
	**Weighted Mean**	**Mean**	**SD**	**Median**	**Min**	**Max**	**Weighted Mean**	**Mean**	**SD**	**Median**	**Min**	**Max**
Ready Meals	0.99	1.24	1.30	1.10	0.00	18.30	0.95	1.25	1.31	1.10	0.00	18.30
S, D & C	2.61	5.14	10.63	1.80	0.00	74.00	2.53	5.13	10.62	1.80	0.00	74.00
Baked Goods	1.02	0.87	0.72	0.90	0.00	13.00	1.13	0.88	0.72	0.90	0.00	13.00
Processed F & V	0.38	0.37	0.69	0.50	0.00	12.50	0.37	0.37	0.70	0.50	0.00	12.50
Processed M & S	2.07	1.72	1.31	1.50	0.00	17.50	2.00	1.72	1.31	1.50	0.00	17.50
R, P & N	0.25	1.36	2.29	1.10	0.00	15.90	0.25	1.37	2.31	1.10	0.00	15.90
Savoury snacks	1.55	1.61	1.21	1.50	0.00	12.00	1.46	1.61	1.21	1.50	0.00	12.00
Soup	1.24	2.79	5.22	0.90	0.00	60.90	1.20	2.56	4.93	0.90	0.00	60.90
**Category**	**Mean Difference (2015–2018)**											
	**Weighted**						**Arithmetic**					
	**g/100 g**		**%**		***p* Value**		**g/100 g**		**%**		***p* Value**	
Ready Meals	−0.04		−4.1%		0.677		0.01		1.1%		0.826	
S, D & C	−0.08		−3.2%		0.917		−0.01		−0.2%		0.976	
Baked Goods	0.11		11.2%		0.000		0.01		0.8%		0.822	
Processed F & V	−0.01		−3.8%		0.520		0.00		−0.9%		0.899	
Processed M & S	−0.06		−3.0%		0.022		0.00		0.1%		0.967	
R, P & N	0.00		1.0%		0.959		0.01		0.6%		0.954	
Savoury snacks	−0.09		−5.6%		0.851		0.01		0.4%		0.873	
Soup	−0.04		−3.2%		0.911		−0.24		−8.4%		0.504	

Source: Elaborated by the authors based on data from Euromonitor (2020). Note: S, D & C = Sauces, Dressings and Condiments. R, P & N = Rice, Pasta and Noodles. SD = standard deviation.

**Table 5:** Summary statistics and variations of fibre content (in g/100 g) for selected packaged food categories in Europe.

**Category**	**2015**						**2018**					
	**Weighted Mean**	**Mean**	**SD**	**Median**	**Min**	**Max**	**Weighted Mean**	**Mean**	**SD**	**Median**	**Min**	**Max**
Break. Cereals	6.7	7.1	4.2	6.8	0.0	38.0	6.6	7.2	4.2	6.9	0.0	38.0
Ready Meals	1.8	1.9	1.8	1.7	0.0	18.0	1.6	1.9	1.8	1.7	0.0	18.0
Sweet Biscuits	3.5	4.3	3.4	3.4	0.0	31.0	3.3	4.4	3.5	3.5	0.0	34.0
Baked Goods	4.9	3.4	3.0	2.7	0.0	27.0	4.9	3.4	3.0	2.7	0.0	27.0
R, P & N	3.0	2.6	1.7	3.0	0.0	15.2	2.9	2.7	1.7	3.0	0.0	15.2
Processed F & V	3.0	2.8	2.2	2.7	0.0	24.9	3.0	2.8	2.1	2.6	0.0	23.0
Savoury snacks	5.3	5.3	4.1	4.2	0.0	29.0	5.1	5.3	4.1	4.2	0.0	29.0
Sweet spreads	2.6	2.8	2.9	2.5	0.0	28.0	2.6	2.9	3.0	2.6	0.0	28.0
Soup	1.1	1.5	2.0	1.0	0.0	19.8	1.1	1.5	2.0	1.0	0.0	19.8
**Category**	**Mean Difference (2015–2018)**											
	**Weighted**						**Arithmetic**					
	**g/100 g**		**%**		***p* Value**		**g/100 g**		**%**		***p* Value**	
Break. Cereals	−0.08		−1.3%		0.553		0.11		1.6%		0.566	
Ready Meals	−0.17		−9.3%		0.015		−0.01		−0.3%		0.954	
Sweet Biscuits	−0.17		−4.8%		0.171		0.13		3.0%		0.314	
Baked Goods	−0.01		−0.1%		0.642		0.02		0.7%		0.870	
R, P & N	−0.06		−1.9%		0.922		0.04		1.5%		0.768	
Processed F & V	−0.06		−2.1%		0.797		−0.02		−0.7%		0.850	
Savoury snacks	−0.17		−3.2%		0.762		0.08		1.5%		0.623	
Sweet spreads	0.02		0.7%		0.670		0.11		3.9%		0.615	
Soup	0.04		3.7%		0.523		−0.01		−0.6%		0.952	

Source: Elaborated by the authors based on data from Euromonitor (2020). Note: R, P & N = Rice, Pasta and Noodles. SD = standard deviation.

**Figure A1:**
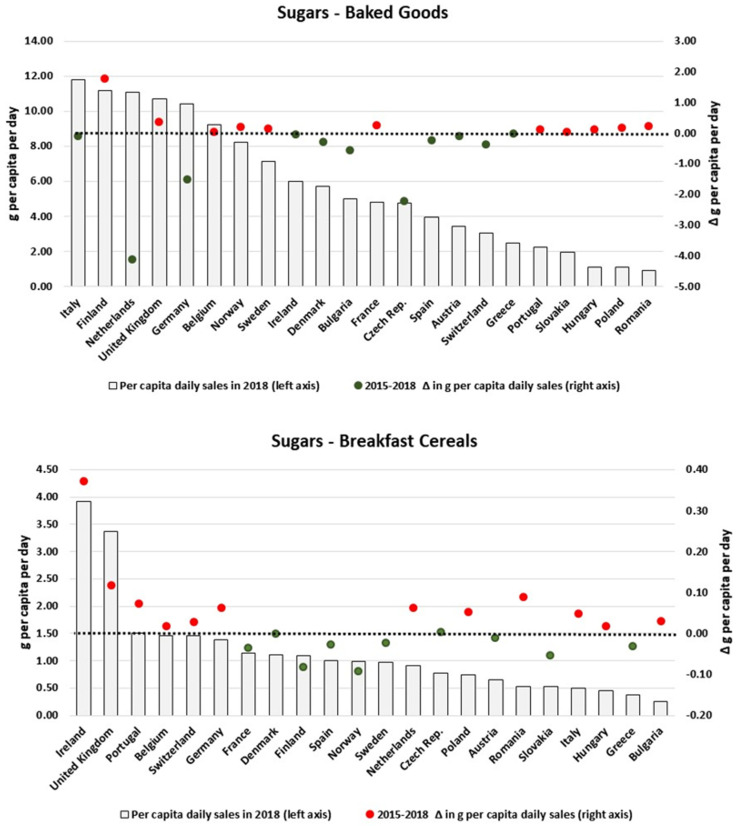
Estimates of total sugars sold through Baked Goods and Breakfast Cereals in 2018 and the respective 2015–2018 variation (in g/pc/day). Source: Elaborated by the authors based on data from Euromonitor (2020) and mid-year population estimates from the HFA-DB. Estimates were scaled by market coverage to represent 100% of the retail market. The right-hand axis represents the nutrient variation in g per capita per day (green for negative values, red for positive values), and the dotted black line indicates the variation of 0 g per capita per day as a reference.

**Figure A2:**
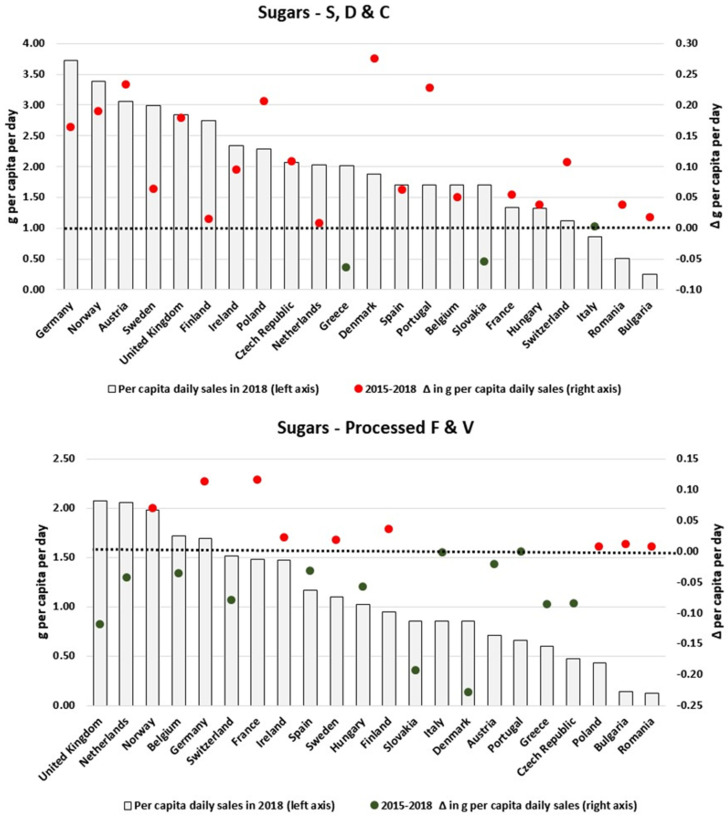
Estimates of total sugars sold through S, D & C and Processed F & V in 2018 and the respective 2015–2018 variation (in g/pc/day). Source: Elaborated by the authors based on data from Euromonitor (2020) and mid-year population estimates from the HFA-DB. Estimates were scaled by market coverage to represent 100% of the retail market. The right-hand axis represents the nutrient variation in g per capita per day (green for negative values, red for positive values), and the dotted black line indicates the variation of 0 g per capita per day as a reference.

**Figure A3:**
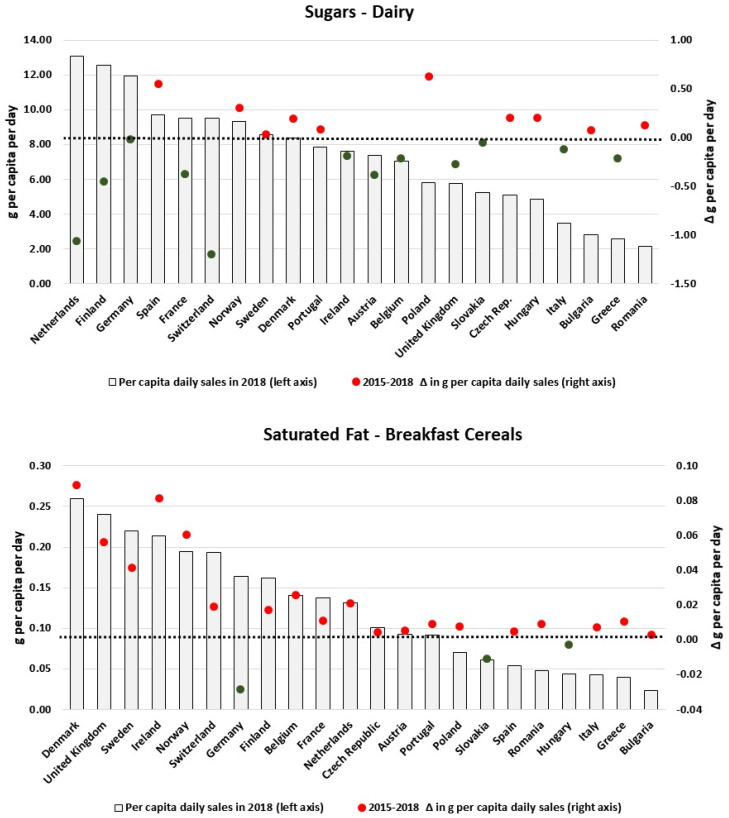
Estimates of total sugars and saturated fat sold through Dairy and Breakfast Cereals respectively, in 2018 and the respective 2015–2018 variation (in g/pc/day). Source: Elaborated by the authors based on data from Euromonitor (2020) and mid-year population estimates from the HFA-DB. Estimates were scaled by market coverage to represent 100% of the retail market. The right-hand axis represents the nutrient variation in g per capita per day (green for negative values, red for positive values), and the dotted black line indicates the variation of 0 g per capita per day as a reference.

**Figure A4:**
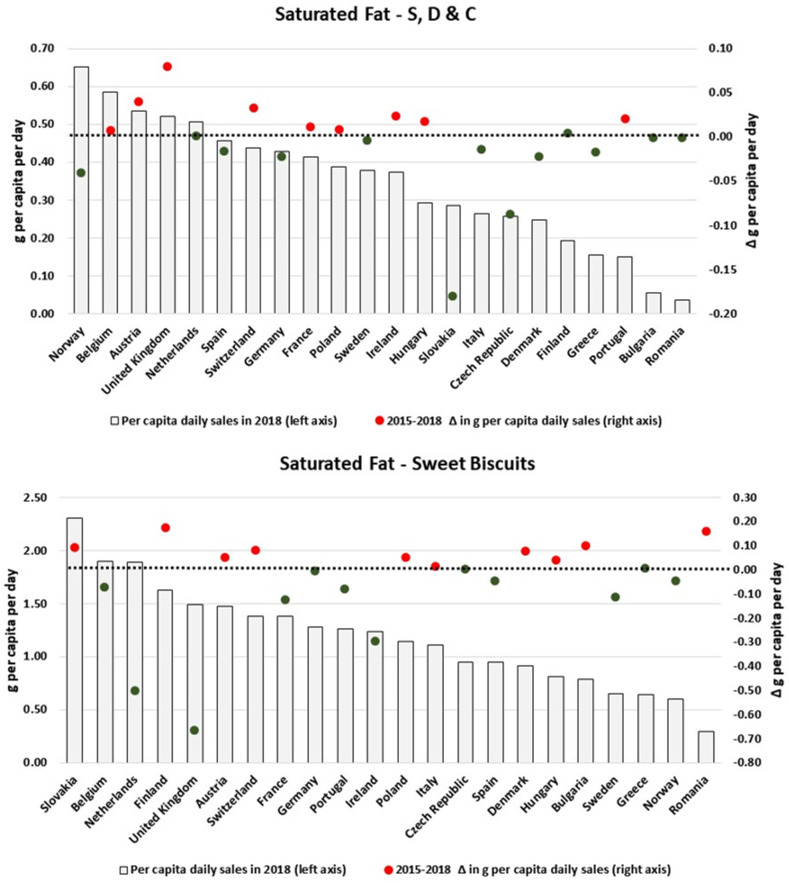
Estimates of saturated fat sold through S, D & C and Sweet Biscuits in 2018 and the respective 2015–2018 variation (in g/pc/day). Source: Elaborated by the authors based on data from Euromonitor (2020) and mid-year population estimates from the HFA-DB. Estimates were scaled by market coverage to represent 100% of the retail market. The right-hand axis represents the nutrient variation in g per capita per day (green for negative values, red for positive values), and the dotted black line indicates the variation of 0 g per capita per day as a reference.

**Figure A5:**
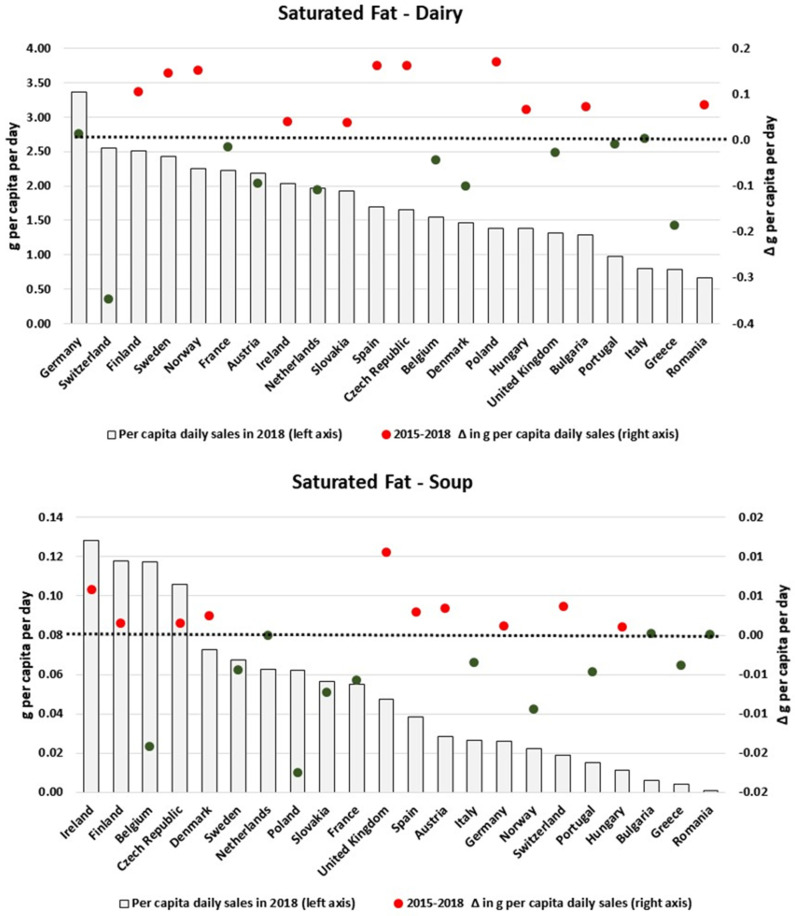
Estimates of saturated fat sold through Dairy and Soup in 2018 and the respective 2015–2018 variation (in g/pc/day). Source: Elaborated by the authors based on data from Euromonitor (2020) and mid-year population estimates from the HFA-DB. Estimates were scaled by market coverage to represent 100% of the retail market. The right-hand axis represents the nutrient variation in g per capita per day (green for negative values, red for positive values), and the dotted black line indicates the variation of 0 g per capita per day as a reference.

**Figure A6:**
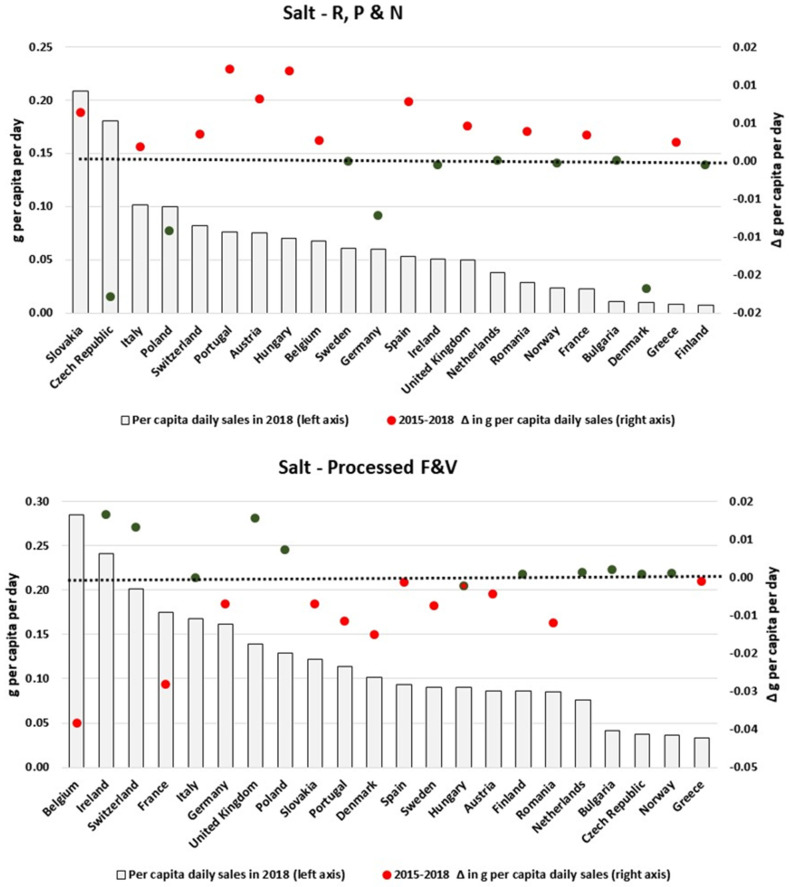
Estimates of salt sold through R, P & N and Processed F & V in 2018 and the respective 2015–2018 variation (in g/pc/day). Source: Elaborated by the authors based on data from Euromonitor (2020) and mid-year population estimates from the HFA-DB. Estimates were scaled by market coverage to represent 100% of the retail market. The right-hand axis represents the nutrient variation in g per capita per day (green for negative values, red for positive values), and the dotted black line indicates the variation of 0 g per capita per day as a reference.

**Figure A7:**
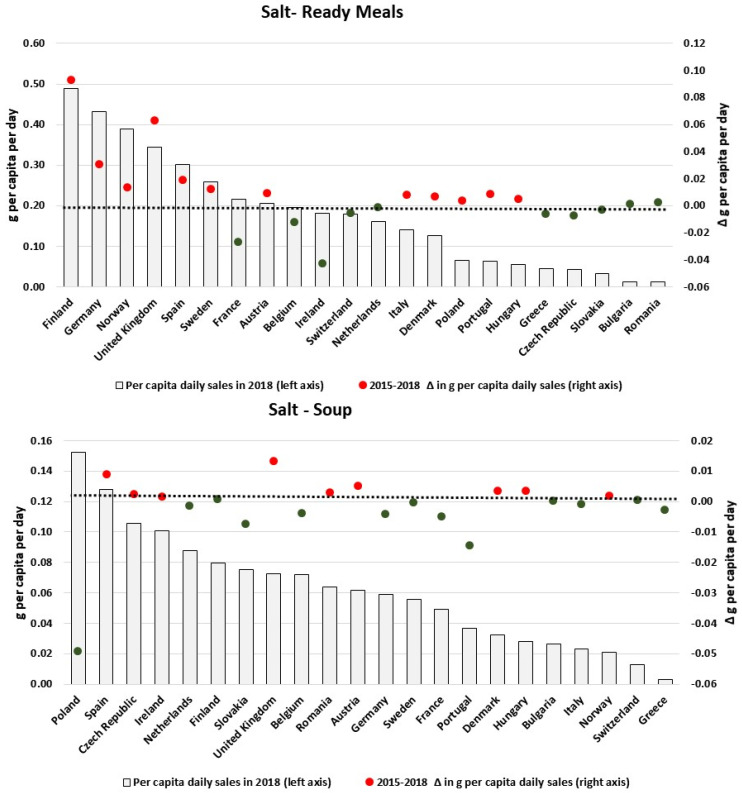
Estimates of salt sold through Ready Meals and Soup in 2018 and the respective 2015–2018 variation (in g/pc/day). Source: Elaborated by the authors based on data from Euromonitor (2020) and mid-year population estimates from the HFA-DB. Estimates were scaled by market coverage to represent 100% of the retail market. The right-hand axis represents the nutrient variation in g per capita per day (green for negative values, red for positive values), and the dotted black line indicates the variation of 0 g per capita per day as a reference.

**Figure A8:**
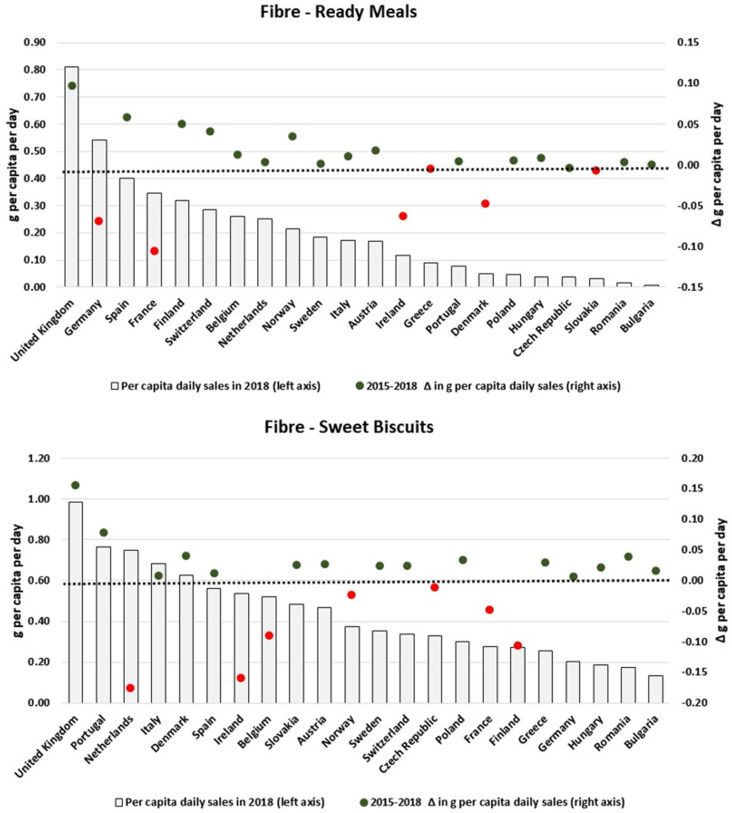
Estimates of fibre sold through Ready Meals and Sweet Biscuits in 2018 and the respective 2015–2018 variation (in g/pc/day). Source: Elaborated by the authors based on data from Euromonitor (2020) and mid-year population estimates from the HFA-DB. Estimates were scaled by market coverage to represent 100% of the retail market. The right-hand axis represents the nutrient variation in g per capita per day (green for positive values, red for negative values), and the dotted black line indicates the variation of 0 g per capita per day as a reference.

**Figure A9:**
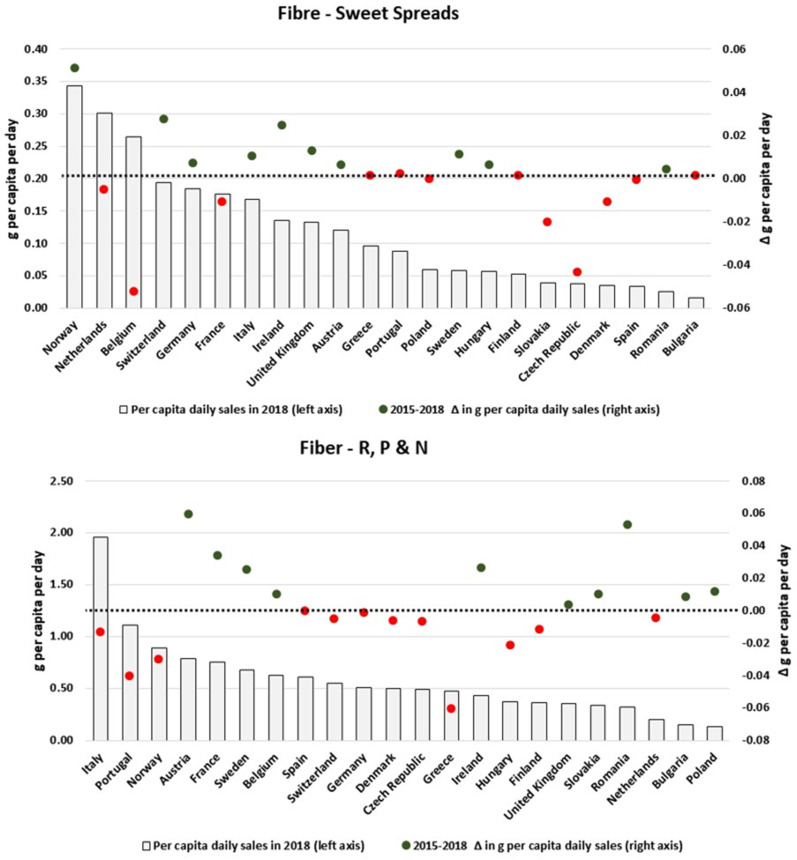
Estimates of fibre sold through Sweet Spreads and R, P & N in 2018 and the respective 2015–2018 variation (in g/pc/day). Source: Elaborated by the authors based on data from Euromonitor (2020) and mid-year population estimates from the HFA-DB. Estimates were scaled by market coverage to represent 100% of the retail market. The right-hand axis represents the nutrient variation in g per capita per day (green for positive values, red for negative values), and the dotted black line indicates the variation of 0 g per capita per day as a reference.

**Figure A10:**
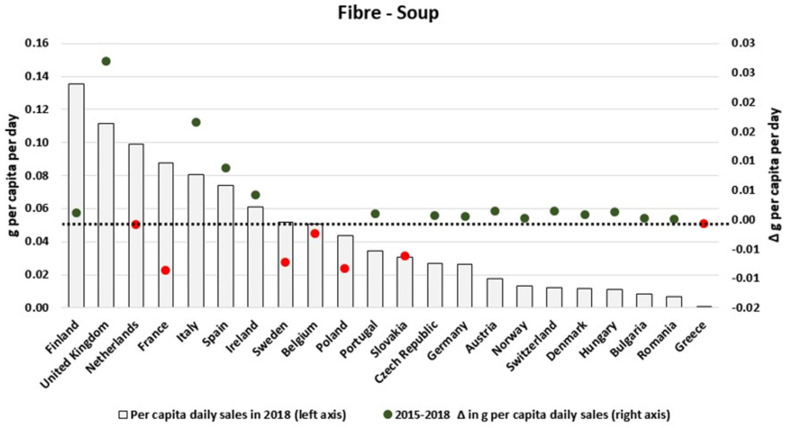
Estimates of fibre sold through Soups in 2018 and the respective 2015–2018 variation (in g/pc/day). Source: Elaborated by the authors based on data from Euromonitor (2020) and mid-year population estimates from the HFA-DB. Estimates were scaled by market coverage to represent 100% of the retail market. The right-hand axis represents the nutrient variation in g per capita per day (green for positive values, red for negative values), and the dotted black line indicates the variation of 0 g per capita per day as a reference.

## Data Availability

The data that support the findings of this study are available from Euromonitor International (https://www.euromonitor.com/, accessed on 8 March 2020), but restrictions apply to the availability of these data, which were used under license for the current study, and so are not publicly available. However, data are available from the authors upon reasonable request and with permission from Euromonitor International. While every attempt has been made to ensure accuracy and reliability, Euromonitor International cannot be held responsible for omissions or errors of historic figures or analyses.

## Appendix A

**Table A1:** Product categorisation and sub-categorisation in the Euromonitor Nutrition database.

Category	Subcategory
Breakfast Cereals	Children’s Breakfast Cereals, Flakes, Hot Cereals, Muslei and Granola, Other RTE Cereals
Soft Drinks	Asian Speciality Drinks, Carbonated RTD Tea, Coconut and Other Plant Waters, Flavoured Bottled Water, Functional Bottled Water, Ginger Ale, Juice Drinks (up to 24% Juice), Lemonade/Lime, Liquid Concentrades, Low Calorie Cola Carbonates, Nectars, Not from Concentrade 100% Juice, Orange Carbonates, Other Non-Cola Carbonates, RTD Coffee, Reconstituted 100% Juice, Regular Cola Carbonates, Regular Energy Drinks, Regular Sports Drinks, Still RTD Tea, Tonic Water/Other Bitters
Dairy	Bulk Dairy Ice Cream, Bulk Water Ice Cream, Chilled Dairy Desserts, Chilled Snacks, Coffee Whiteners, Dairy-Only Flavoured Milk Drinks, Drinking Yoghurt, Flavoured Fromage Frais and Quark, Flavoured Milk Drinks with Fruit Juice, Flavoured Yoghurt, Frozen Yoghurt, Ice Cream Desserts, MultiPack Dairy Ice Cream, MultiPack Water Ice Cream, Plain Fromage Frais and Quark, Plain Yoghurt, Savoury Fromage Frais and Quark, Shelf-Stable Dairy Desserts, Single-Portion Dairy Ice Cream, Single-Portion Water Ice Cream, Sour Milk Products, Soy Drinks
Ready Meals	Chilled Pizza, Chilled Ready Meals, Dinner Mixes, Dried Ready Meals, Frozen Pizza, Frozen Ready Meals, Shelf-Stable Ready Meals
S, D & C	Barbecue Sauces, Chili Sauces, Cooking Sauces, Dips, Dry Sauces, Fish Sauces, Gravy Cubes and Powders, Ketchup, Liquid Stocks and Fons, Mayonnaise, Monosodium Glutamate, Mustard, Other Sauces, Dressings and Condiments, Other Table Sauces, Oyster Sauces, Pasta Sauces, Pickled Products, Salad Dressings, Soy Sauces, Stock Cubes and Powders, Tomato Pastes and Purées, Yeast-based Spreads
Soup	Chilled Soup, Dehydrated Soup, Frozen Soup, Instant Soup, Shelf-Stable Soup
Sweet Spreads	Chocolate Spreads, Jams and Preserves, Nut- and Seed-Based Spreads
Confectionary	Boiled Sweets, Boxed Assortments, Bubble Gum, Chocolate Pouches and Bags, Chocolate with Toys, Countlines, Liquorice, Lollipops, Medicated Confectionery, Other Chocolate Confectionery, Other Sugar Confectionery, Pastilles, Power Mints, Standard Mints, Tablets, Toffees, Caramels and Nougat
Savoury Snacks	Nuts, Seeds and Trail Mixes, Other Savoury Snacks, Popcorn, Potato Chips, Pretzels, Puffed Snacks, Rice Snacks, Savoury Biscuits, Tortilla Chips, Vegetable, Pulse and Bread Chips
Sweet Biscuits	Cereal Bars, Chocolate Coated Biscuits, Cookies, Dried Fruit, Energy Bars, Filled Biscuits, Fruit and Nut Bars, Other Snack Bars, Plain Biscuits, Processed Fruit Snacks, Wafers
Baked Goods	Dessert Mixes, Frozen Baked Goods, Packaged Cakes, Packaged Flat Bread, Packaged Leavened Bread, Packaged Pastries
Processed M & S	Chilled Meat Substitutes, Chilled Processed Poultry, Chilled Processed Red Meat, Chilled Processed Seafood, Frozen Meat Substitutes, Frozen Processed Poultry, Frozen Processed Red Meat, Frozen Processed Seafood, Shelf Stable Meat Substitutes, Shelf-Stable Processed Poultry, Shelf Stable Processed Red Meat, Shelf-Stable Seafood
R, P & N	Chilled Noodles, Chilled Pasta, Dried Pasta, Instant Noodle Cups, Instant Noodle Pouches
Processed F & V	Frozen Fruit, Frozen Processed Potatoes, Frozen Processed Vegetables, Shelf-Stable Beans, Shelf-Stable Fruit, Shelf-Stable Tomatoes, Shelf-Stable Vegetables

Source: Elaborated by the authors based on data from Euromonitor (2020). Note: RTD, ready-to-drink; RTE, ready-to-eat.

**Table A2:** Number of products extracted from the Euromonitor database and their respective market coverage (in %) in 2015, per category and country.

	**Break. Cer.**		**Dairy**		**Soft Drinks**		**Ready Meals**		**S, D & C**		**Soups**		**Sweet Spr.**	
		**% Cov.**		**% Cov.**		**% Cov.**		**% Cov.**		**% Cov.**		**% Cov.**		**% Cov.**
Austria	25	73%	111	60%	148	77%	34	80%	83	83%	16	86%	24	73%
Belgium	45	73%	166	79%	107	66%	49	65%	117	78%	22	55%	28	72%
Bulgaria	39	59%	121	46%	138	79%	19	74%	111	77%	16	92%	26	37%
Czech Republic	50	83%	197	81%	148	76%	29	57%	126	61%	18	88%	18	34%
Denmark	44	71%	129	87%	147	89%	25	71%	104	69%	14	83%	22	58%
Finland	42	87%	151	86%	185	75%	42	89%	116	92%	24	93%	15	63%
France	52	72%	158	67%	153	77%	54	54%	106	72%	19	80%	34	66%
Germany	43	69%	176	73%	155	65%	51	72%	105	77%	17	84%	22	62%
Greece	30	77%	125	71%	98	85%	42	82%	101	87%	3	29%	19	47%
Hungary	63	82%	251	78%	236	82%	31	73%	125	71%	6	70%	29	46%
Ireland	57	64%	134	67%	94	76%	33	37%	108	75%	18	83%	26	56%
Italy	35	83%	151	73%	128	70%	70	43%	121	81%	20	73%	26	77%
Netherlands	36	57%	136	79%	114	63%	54	75%	151	81%	17	88%	25	71%
Norway	44	94%	95	92%	130	88%	25	71%	96	84%	13	94%	26	87%
Poland	61	83%	163	68%	184	76%	48	76%	130	83%	27	70%	28	62%
Portugal	44	69%	92	77%	80	54%	30	66%	63	52%	8	22%	16	41%
Romania	47	50%	153	71%	105	66%	20	57%	122	69%	8	61%	21	67%
Slovakia	41	86%	146	79%	181	77%	28	57%	106	68%	18	67%	19	34%
Spain	33	70%	144	72%	120	87%	48	54%	127	67%	29	79%	23	59%
Sweden	64	84%	143	82%	158	81%	49	82%	177	90%	27	86%	26	66%
Switzerland	42	71%	161	78%	112	79%	27	65%	66	86%	15	96%	12	58%
United Kingdom	60	83%	156	74%	161	84%	61	72%	109	65%	35	82%	27	67%
Total	997	74%	3259	75%	3082	76%	869	67%	2470	76%	390	76%	512	59%
	**Confec.**		**Savoury Sn.**		**Sweet Bisc.**		**Baked Goods**		**Proc. M & S**		**R, P & N**		**Proc. F & V**	
		**% Cov.**		**% Cov.**		**% Cov.**		**% Cov.**		**% Cov.**		**% Cov.**		**% Cov.**
Austria	122	79%	63	75%	69	63%	30	47%	64	59%	15	53%	56	74%
Belgium	146	67%	56	70%	77	63%	57	49%	80	66%	21	61%	48	78%
Bulgaria	149	81%	86	78%	138	78%	58	36%	63	85%	15	34%	65	81%
Czech Republic	141	76%	87	78%	97	80%	53	29%	61	61%	33	38%	54	61%
Denmark	122	65%	54	83%	61	80%	38	37%	77	74%	14	30%	51	52%
Finland	138	68%	70	77%	63	49%	52	55%	79	95%	22	51%	59	91%
France	122	43%	76	76%	85	65%	57	61%	95	65%	22	46%	53	71%
Germany	175	68%	65	75%	83	57%	39	45%	75	64%	23	68%	37	71%
Greece	98	80%	46	73%	60	80%	52	63%	55	70%	18	75%	43	78%
Hungary	164	64%	80	67%	102	69%	84	75%	92	69%	36	49%	66	64%
Ireland	130	65%	83	72%	56	48%	55	44%	32	70%	18	35%	20	63%
Italy	138	66%	90	67%	106	81%	94	42%	149	63%	49	62%	102	68%
Netherlands	145	71%	63	67%	62	47%	36	44%	73	73%	15	24%	48	76%
Norway	78	60%	52	79%	45	76%	47	62%	56	85%	13	33%	37	82%
Poland	160	80%	94	80%	106	81%	52	71%	75	67%	37	53%	75	84%
Portugal	91	63%	54	86%	67	68%	35	64%	63	78%	19	35%	37	75%
Romania	118	69%	71	76%	116	72%	41	61%	82	68%	21	21%	65	70%
Slovakia	129	71%	86	72%	101	78%	56	51%	49	69%	26	36%	39	46%
Spain	147	58%	71	74%	94	62%	48	69%	88	62%	29	39%	46	73%
Sweden	120	66%	98	80%	78	79%	59	58%	128	86%	32	40%	75	88%
Switzerland	119	56%	65	83%	66	54%	24	71%	33	78%	17	67%	25	87%
United Kingdom	161	60%	95	59%	78	43%	79	58%	79	77%	27	29%	57	74%
Total	2913	67%	1605	75%	1810	67%	1146	54%	1648	72%	522	44%	1158	73%

Source: Elaborated by the authors based on data from Euromonitor (2020). Note: S, D & C = Sauces, Dressings and Condiments; Processed M & S = Processed Meat and Seafood; R, P & N = Rice, Pasta and Noodles; Processed F & V = Processed Fruits and Vegetables.

**Table A3:** Changes in the market-share weighted mean of total sugars (in g/100 g and %) between 2015 and 2018.

Country	Soft Drinks		Br. Cereals		Dairy		Proc. F & V		Baked Goods		Sweet Biscuits		Sweet Spreads		Confec.		Sauces	
	g/100 g	%	g/100 g	%	g/100 g	%	g/100 g	%	g/100 g	%	g/100 g	%	g/100 g	%	g/100 g	%	g/100 g	%
Austria	−0.1	−2%	−1.1	−6%	−0.7	−6%	−0.2	−6%	−0.7	−10%	−0.2	−1%	0.4	1%	0.0	0%	−0.2	−2%
Belgium	−0.4	−6%	−0.8	−4%	−0.2	−2%	0.1	4%	0.1	0%	1.1	3%	−7.9	−15%	−1.3	−3%	0.1	1%
Bulgaria	0.4	4%	−0.8	−4%	−0.1	−2%	0.0	2%	0.3	5%	−0.3	−1%	−0.9	−2%	−0.3	−1%	−0.7	−9%
Czech Rep.	0.5	6%	−0.1	−1%	0.5	5%	−0.3	−10%	−0.1	−2%	0.1	0%	−11.8	−23%	−1.5	−3%	−0.6	−6%
Denmark	−0.3	−5%	−0.2	−4%	−0.3	−4%	−1.3	−38%	−0.7	−13%	−0.5	−1%	−4.8	−11%	−4.3	−9%	1.8	21%
Finland	−0.3	−5%	−0.5	−6%	−0.1	−2%	0.1	4%	0.7	9%	0.6	2%	−0.8	−2%	−0.7	−1%	0.0	0%
France	−0.4	−4%	−1.3	−6%	−0.1	−1%	0.5	16%	0.1	1%	0.3	1%	−1.9	−4%	−7.8	−14%	0.1	2%
Germany	−0.1	−2%	0.0	0%	−0.2	−2%	0.3	7%	0.4	6%	−0.5	−1%	1.6	3%	−0.6	−1%	0.2	2%
Greece	−0.2	−3%	0.0	0%	0.4	5%	−0.2	−3%	0.9	13%	−1.2	−4%	−2.0	−4%	−5.6	−12%	0.2	3%
Hungary	−0.2	−3%	−0.3	−1%	−0.2	−1%	−0.5	−12%	−0.4	−6%	−0.2	−1%	−1.4	−3%	−0.6	−1%	0.0	0%
Ireland	−0.2	−2%	−0.9	−6%	−0.6	−4%	−0.1	−4%	0.0	0%	0.1	0%	−9.5	−21%	−0.5	−1%	0.1	1%
Italy	−0.2	−2%	−0.1	−1%	−0.6	−5%	−0.1	−2%	−0.7	−3%	0.2	1%	−0.5	−1%	−1.9	−4%	−0.2	−4%
Netherlands	−0.5	−5%	−0.5	−4%	−0.5	−5%	−0.2	−3%	0.0	0%	−0.1	0%	0.7	2%	−0.9	−2%	−0.2	−2%
Norway	−1.0	−15%	0.1	1%	−0.1	−1%	−0.3	−9%	0.0	0%	1.7	6%	0.5	1%	−4.3	−8%	0.0	0%
Poland	−0.5	−6%	−0.2	−1%	0.3	3%	−0.2	−9%	0.8	14%	−0.2	−1%	−1.5	−3%	−0.5	−1%	0.3	3%
Portugal	1.3	16%	−0.3	−1%	0.6	6%	0.4	11%	−1.0	−9%	0.2	1%	−20.8	−44%	−1.6	−3%	−0.3	−4%
Romania	−0.8	−8%	0.5	2%	−0.3	−5%	−1.7	−26%	0.1	3%	0.5	2%	0.1	0%	−1.0	−2%	−0.4	−4%
Slovakia	−0.3	−4%	−1.6	−7%	−0.3	−3%	0.0	0%	0.1	1%	1.1	3%	−8.7	−16%	−0.3	0%	−0.4	−5%
Spain	−0.2	−2%	−0.6	−3%	0.9	9%	0.0	−1%	−1.7	−11%	−0.1	0%	−0.1	0%	−1.5	−3%	0.2	3%
Sweden	−0.9	−11%	−0.7	−9%	0.0	1%	−0.3	−6%	0.2	3%	−0.2	0%	−1.7	−4%	0.5	1%	0.2	2%
Switzerland	−0.2	−3%	−0.4	−2%	−1.0	−10%	−0.1	−3%	−1.2	−13%	1.0	3%	−0.2	0%	−0.7	−1%	0.4	6%
UK	−0.3	−5%	−1.2	−9%	−0.7	−6%	0.0	1%	0.4	5%	−0.4	−1%	−3.7	−8%	−0.7	−1%	−0.3	−3%

Source: Elaborated by the authors based on data from Euromonitor (2020).

**Table A4:** Changes in the market-share weighted mean of saturated fat (in g/100 g and %) between 2015 and 2018.

Country	Break. Cereals		Ready Meals		S, D &C		Sweet Biscuits		Baked Goods		Proc. M&S		Dairy		Soup		Savoury Snacks	
	g/100 g	%	g/100 g	%	g/100 g	%	g/100 g	%	g/100 g	%	g/100 g	%	g/100 g	%	g/100 g	%	g/100 g	%
Austria	0.0	1%	−0.1	−3%	0.0	−2%	−0.2	−2%	0.1	8%	0.1	1%	−0.2	−6%	0.0	3%	−0.1	−3%
Belgium	0.1	4%	−0.1	−5%	0.0	−1%	−0.4	−5%	0.0	1%	0.0	−2%	0.0	−2%	−0.1	−6%	0.0	0%
Bulgaria	−0.1	−4%	0.0	−1%	−0.4	−20%	0.2	2%	0.1	4%	−0.2	−3%	0.1	6%	0.0	0%	0.0	0%
Czech Rep.	0.1	4%	−0.5	−18%	−0.3	−21%	−0.1	−1%	−0.1	−8%	−0.4	−10%	0.3	10%	−0.3	−4%	0.3	7%
Denmark	0.3	42%	−0.2	−9%	0.0	−1%	0.2	4%	0.0	−3%	0.3	6%	−0.2	−12%	0.0	2%	−0.2	−6%
Finland	−0.1	−5%	0.0	0%	0.0	2%	1.1	12%	0.2	10%	−0.1	−4%	0.1	7%	0.0	0%	0.4	11%
France	0.1	3%	−0.4	−14%	0.0	1%	−0.4	−4%	0.2	7%	−0.2	−5%	0.1	2%	0.0	−1%	−0.2	−4%
Germany	−0.6	−19%	−0.1	−3%	−0.1	−7%	−0.5	−4%	0.1	3%	−0.3	−6%	0.0	−1%	0.1	7%	−0.8	−15%
Greece	0.3	28%	0.4	13%	0.0	−4%	−0.2	−2%	−0.3	−8%	−0.3	−9%	−0.2	−8%	0.1	4%	0.6	8%
Hungary	−0.3	−12%	0.1	7%	0.1	3%	0.1	1%	0.1	6%	−1.0	−11%	0.0	−1%	0.0	−2%	−0.6	−8%
Ireland	0.1	9%	−0.6	−21%	0.1	4%	−1.8	−20%	−0.1	−11%	0.1	2%	0.0	0%	0.1	3%	0.0	−1%
Italy	0.1	6%	0.0	2%	−0.1	−4%	−0.1	−1%	−0.2	−4%	−0.1	−3%	0.0	−1%	−0.2	−23%	−0.2	−5%
Netherlands	0.1	5%	−0.2	−7%	−0.1	−2%	−1.9	−20%	−0.3	−20%	−1.1	−19%	0.0	−2%	0.0	1%	−0.2	−4%
Norway	0.1	7%	0.0	0%	−0.3	−9%	−0.2	−3%	0.0	0%	0.0	0%	0.1	2%	−0.2	−17%	−0.1	−1%
Poland	0.1	4%	0.0	−2%	−0.1	−4%	0.0	0%	0.0	1%	−1.3	−19%	0.1	5%	−1.0	−37%	−0.6	−8%
Portugal	0.0	0%	−0.3	−9%	0.0	−1%	−0.5	−6%	−0.3	−12%	−0.1	−2%	0.0	4%	−0.4	−28%	−0.4	−7%
Romania	0.1	6%	0.8	42%	−0.1	−15%	0.7	8%	−0.2	−6%	−0.5	−8%	0.0	1%	0.0	−3%	−0.1	−1%
Slovakia	−0.4	−13%	−0.7	−23%	−0.8	−36%	0.1	1%	0.0	4%	−0.7	−16%	0.1	3%	−0.1	−3%	0.1	1%
Spain	0.1	6%	−0.1	−2%	−0.1	−4%	−0.2	−3%	−0.4	−7%	0.0	−1%	0.2	13%	0.0	−9%	−0.1	−3%
Sweden	0.2	12%	−0.1	−3%	0.0	−1%	−1.6	−19%	0.0	−2%	−0.2	−3%	0.1	7%	0.0	−1%	0.0	−1%
Switzerland	0.1	3%	0.2	8%	0.1	3%	0.8	7%	−0.3	−7%	0.1	3%	−0.3	−11%	0.2	19%	0.0	0%
UK	0.0	1%	0.1	3%	0.1	7%	−2.7	−30%	0.1	5%	−0.3	−8%	−0.1	−2%	0.0	4%	0.0	−1%

Source: Elaborated by the authors based on data from Euromonitor (2020).

**Table A5:** Changes in the market-share weighted mean of salt (in g/100 g and %) between 2015 and 2018.

Country	Ready Meals		S, D &C		Baked Goods		Proc. M & S		R, P & N		Savoury Snacks		Soup		Processed F&V	
	g/100 g	%	g/100 g	%	g/100 g	%	g/100 g	%	g/100 g	%	g/100 g	%	g/100 g	%	g/100 g	%
Austria	−0.1	−4%	0.0	−2%	0.0	−3%	−0.1	−3%	0.0	3%	−0.1	−4%	0.0	−1%	0.0	−8%
Belgium	−0.1	−8%	0.0	−1%	0.0	−3%	0.0	0%	0.0	3%	0.1	3%	0.0	0%	0.0	−5%
Bulgaria	0.0	0%	−0.4	−11%	0.0	−3%	0.1	5%	0.0	−2%	−0.1	−4%	−0.3	−3%	0.0	−2%
Czech Rep.	−0.3	−23%	−0.9	−29%	0.0	3%	−0.4	−19%	0.0	1%	−0.1	−6%	−0.2	−3%	0.0	−5%
Denmark	0.0	−4%	−0.6	−20%	0.0	−4%	0.0	3%	−0.1	−35%	−0.1	−6%	0.1	8%	0.0	−11%
Finland	0.0	−2%	0.0	−1%	−0.1	−9%	0.0	2%	0.0	−10%	0.0	0%	0.0	−1%	0.0	1%
France	−0.1	−11%	−0.1	−4%	−0.1	−9%	0.0	0%	0.0	5%	−0.1	−7%	0.0	−1%	0.0	0%
Germany	0.0	2%	0.0	−1%	0.3	27%	−0.1	−5%	0.0	−4%	−0.2	−11%	−0.1	−4%	0.0	−5%
Greece	−0.1	−15%	0.1	13%	0.1	9%	−0.2	−9%	0.0	52%	0.0	−1%	−0.1	−1%	0.0	3%
Hungary	−0.1	−6%	−0.2	−5%	−0.1	−8%	−0.3	−10%	0.0	18%	0.0	0%	0.1	2%	0.0	−8%
Ireland	−0.2	−24%	0.0	−2%	0.0	−3%	0.1	4%	0.0	−5%	0.0	−3%	0.0	0%	0.0	1%
Italy	0.0	−3%	−0.1	−7%	0.0	1%	0.0	1%	0.0	2%	0.0	−2%	−0.1	−16%	0.0	2%
Netherlands	0.0	−4%	−0.1	−3%	0.0	−2%	−0.3	−16%	0.0	−2%	0.0	0%	0.0	0%	0.0	−5%
Norway	0.0	−5%	0.1	3%	0.0	1%	0.0	−1%	0.0	3%	0.0	−1%	0.2	30%	0.0	−7%
Poland	0.0	−4%	−0.1	−3%	0.0	−1%	−0.2	−11%	−0.1	−12%	−0.1	−7%	−2.7	−41%	0.0	−2%
Portugal	0.0	4%	0.2	8%	−0.1	−12%	0.0	2%	0.0	23%	−0.1	−10%	−1.2	−32%	−0.1	−8%
Romania	0.0	3%	−0.4	−8%	0.0	0%	0.2	7%	0.0	−2%	0.1	3%	−0.2	−2%	−0.1	−13%
Slovakia	−0.2	−14%	−0.8	−20%	0.0	−1%	−0.3	−13%	0.0	4%	0.1	6%	0.0	0%	0.0	−3%
Spain	0.0	−3%	−0.1	−5%	−0.1	−7%	0.0	1%	0.0	17%	0.0	−4%	−0.1	−10%	0.0	−1%
Sweden	0.0	−1%	0.0	0%	0.0	2%	−0.1	−4%	0.0	−4%	−0.1	−4%	0.0	5%	0.0	−9%
Switzerland	−0.1	−11%	0.0	1%	0.1	7%	0.0	4%	0.0	5%	0.0	−2%	0.0	0%	0.0	−11%
UK	0.0	−7%	0.0	1%	0.0	1%	−0.1	−6%	0.0	6%	−0.2	−11%	0.0	−1%	0.0	−4%

Source: Elaborated by the authors based on data from Euromonitor (2020).

**Table A6:** Changes in the market-share weighted mean of fibre (in g/100 g and %) between 2015 and 2018.

Country	Break. Cereals		Ready Meals		Sweet Biscuits		Baked Goods		R, P & N		Proc. F&V		Savoury Snacks		Sweet Spread		Soup	
	g/100 g	%	g/100 g	%	g/100 g	%	g/100 g	%	g/100 g	%	g/100 g	%	g/100 g	%	g/100 g	%	g/100 g	%
Austria	0.0	1%	−0.1	−2%	0.0	0%	−0.1	−4%	0.0	0%	−0.4	−12%	−0.1	−2%	0.0	1%	0.0	−1%
Belgium	0.0	1%	0.2	2%	−0.2	−5%	0.1	3%	0.0	1%	−0.1	−2%	−0.2	−5%	−0.5	−17%	0.0	1%
Bulgaria	−0.2	−7%	0.1	1%	0.0	1%	0.4	6%	0.0	0%	0.3	8%	−0.2	−6%	0.0	2%	0.0	0%
Czech Rep.	−0.8	−33%	−0.3	−5%	0.1	2%	0.2	3%	−0.2	−6%	−0.3	−8%	−0.1	−3%	0.0	−1%	0.2	6%
Denmark	−0.3	−42%	−0.6	−7%	0.0	1%	−0.5	−8%	−0.4	−12%	−0.5	−18%	−1.0	−16%	0.0	−4%	0.0	7%
Finland	0.0	−5%	0.2	2%	0.2	9%	−0.9	−14%	−0.1	−6%	0.0	0%	−0.6	−8%	0.0	0%	0.0	0%
France	−0.2	−11%	−0.2	−3%	−0.1	−2%	−0.2	−5%	0.0	−1%	0.0	−2%	−0.2	−4%	0.0	1%	0.0	0%
Germany	−0.3	−16%	0.0	0%	0.0	0%	0.0	−1%	−0.2	−7%	−0.1	−3%	−0.2	−3%	0.0	1%	0.1	5%
Greece	−0.2	−9%	0.7	10%	0.4	12%	−0.1	−3%	−0.1	−5%	0.1	2%	0.0	1%	0.1	4%	−0.1	−3%
Hungary	−0.3	−13%	0.3	5%	0.2	8%	0.2	6%	−0.3	−6%	−0.2	−5%	−0.2	−3%	0.2	8%	0.0	1%
Ireland	0.0	−4%	0.3	6%	−0.8	−22%	−0.1	−2%	0.1	3%	0.1	2%	−0.1	−3%	0.3	10%	0.0	6%
Italy	−0.1	−3%	−0.1	−1%	−0.1	−1%	0.1	4%	0.0	0%	−0.1	−4%	0.0	0%	0.0	0%	0.2	9%
Netherlands	0.0	−1%	0.2	3%	−0.6	−15%	−0.5	−6%	0.0	1%	0.0	0%	0.2	4%	0.0	−1%	0.0	1%
Norway	0.0	−1%	0.7	9%	−0.1	−2%	−0.1	−2%	−0.2	−4%	0.1	3%	0.3	5%	0.2	6%	0.0	3%
Poland	0.0	−4%	0.2	3%	0.2	7%	−0.8	−18%	−0.1	−2%	−0.1	−2%	−0.4	−7%	−0.1	−2%	−1.8	−50%
Portugal	−0.1	−6%	0.6	10%	0.4	10%	−1.1	−26%	0.0	−1%	0.2	8%	−0.1	−2%	0.2	7%	1.6	66%
Romania	0.1	3%	−0.2	−5%	0.0	0%	−0.2	−4%	0.0	1%	−0.8	−19%	−0.3	−6%	0.0	0%	−0.1	−5%
Slovakia	0.1	3%	−0.2	−3%	0.1	2%	0.0	1%	0.0	1%	0.1	3%	−0.4	−9%	0.0	−1%	0.0	1%
Spain	0.1	4%	0.2	3%	0.1	3%	0.0	0%	0.0	−1%	0.0	−2%	0.4	7%	0.0	0%	0.0	−3%
Sweden	0.0	−5%	−0.2	−2%	0.1	2%	−0.1	−2%	0.0	−1%	0.0	0%	−0.2	−2%	0.2	19%	−0.1	−8%
Switzerland	0.1	9%	0.0	0%	0.3	9%	0.1	3%	0.0	−1%	−0.1	−2%	0.4	7%	0.3	13%	0.1	10%
UK	−0.2	−8%	−0.3	−5%	−0.7	−18%	0.0	1%	0.0	−1%	−0.1	−2%	−0.4	−11%	0.2	6%	0.0	5%

Source: Elaborated by the authors based on data from Euromonitor (2020).
